# Guidelines and protocols for cardiovascular magnetic resonance in children and adults with congenital heart disease: SCMR expert consensus group on congenital heart disease

**DOI:** 10.1186/1532-429X-15-51

**Published:** 2013-06-13

**Authors:** Sohrab Fratz, Taylor Chung, Gerald F Greil, Margaret M Samyn, Andrew M Taylor, Emanuela R Valsangiacomo Buechel, Shi-Joon Yoo, Andrew J Powell

**Affiliations:** 1 Department of Pediatric Cardiology and Congenital Heart Disease, Deutsches Herzzentrum München (German Heart Center Munich) of the Technical University Munich, Munich, Germany; 2 Department of Diagnostic Imaging, Children’s Hospital & Research Center Oakland, Oakland, California, USA; 3 Department of Pediatric Cardiology, Evelina Children’s Hospital/Guy’s and St. Thomas’ Hospital NHS Foundation Trust; Division of Imaging Sciences & Biomedical Engineering, King’s College London, London, UK; 4 The Herma Heart Center, Children’s Hospital of Wisconsin, Medical College of Wisconsin, Milwaukee, WI, USA; 5 Centre for Cardiovascular Imaging, UCL Institute of Cardiovascular Science, & Great Ormond Street Hospital for Children, London, UK; 6 Division of Cardiology, University Children’s Hospital Zurich, Zurich, Switzerland; 7 Department of Diagnostic Imaging and Division of Cardiology, Department of Paediatrics, Hospital for Sick Children, University of Toronto, Toronto, Ontario, Canada; 8 Department of Cardiology, Boston Children’s Hospital, and the Department of Pediatrics, Harvard Medical School, Boston, MA, USA

**Keywords:** Cardiovascular magnetic resonance, Congenital heart disease, Heart defects, Imaging protocols, Magnetic resonance imaging

## Abstract

Cardiovascular magnetic resonance (CMR) has taken on an increasingly important role in the diagnostic evaluation and pre-procedural planning for patients with congenital heart disease. This article provides guidelines for the performance of CMR in children and adults with congenital heart disease. The first portion addresses preparation for the examination and safety issues, the second describes the primary techniques used in an examination, and the third provides disease-specific protocols. Variations in practice are highlighted and expert consensus recommendations are provided. Indications and appropriate use criteria for CMR examination are not specifically addressed.

## Introduction

Over the past two decades, there has been a marked increase in the use of cardiovascular magnetic resonance (CMR) for the anatomical and functional evaluation of congenital heart disease (CHD) [1-6]. CMR is rarely used as the initial or sole diagnostic imaging modality. Rather it complements echocardiography, provides a non-invasive alternative to x-ray angiography, avoids the ionizing radiation exposure of computed tomography, and overcomes many of the limitations of these modalities. Expert recommendations regarding the indications for CMR in adults with CHD have recently been published [7] and are underway for children with CHD. This document focuses on the performance of CMR in children and adults with CHD. The first portion addresses preparation for the examination and safety issues, the second describes the primary techniques or modules of an examination, and the third provides disease-specific protocols using these modules. The aim is to provide an educational resource for those engaged in CMR in this patient population and to help standardize the approach to such patients. As much as possible we try to support our recommendations by published evidence. However, where data is lacking, the recommendations represent an expert consensus view.

### Examination preparation and safety

When applicable, parents should be provided with a detailed description of the CMR examination and asked to discuss it with their child in an age-appropriate manner in advance to increase the likelihood of a successful study. Prior to bringing the patient into the scanner room, the physician and technologists should review the patient’s history and safety screening form to identify implanted devices which may be hazardous in the CMR environment or produce image artifact. For patients who may have undergone a cardiac procedure and have an incomplete or unreliable history, a chest radiograph should be obtained to assist screening. A detailed discussion on CMR safety and device interactions can be found elsewhere [8-10].

Following safety screening, physiological monitoring devices and hearing protection (for both awake and sedated patients) are put in place. Young children dissipate body heat faster than adults; thus, patient temperature should be monitored and blankets applied as needed to minimize heat loss. A high-quality electrocardiogram (ECG) signal is essential for optimum data quality in cardiac-gated sequences. The adequacy of the signal should be checked not only at the onset when the patient is outside the scanner bore, but also once inside it and during actual scanning. In patients with dextrocardia, ECG leads are best placed on the right chest.

The imaging coil should be chosen to maximize the signal-to-noise ratio over the body region to be examined. Because CHD often involves abnormalities of the thoracic vasculature, the coil will usually need to be large enough to cover the entire thorax and upper abdomen rather than just the heart. In smaller patients, pediatric thoracic coils or coils designed for an adult-sized head, shoulder, or knee may be suitable. Adequate coil coverage and placement should be confirmed early in the examination by reviewing the localizing images.

#### *Sedation*

Patients undergoing CMR must remain still in the scanner for up to 60 minutes to minimize motion artifact during image acquisition and allow planning of successive imaging sequences. Young children (typically less than age 6 to 8 years) and cognitively impaired older patients typically require some form of sedation. Multiple factors should be taken into account when deciding whether a patient should have an examination with sedation including the length of the anticipated examination protocol, developmental maturity, the patient’s experience with prior procedures, the parents’ opinion of their child’s capability to cooperate with the examination, the risks of sedation, and the benefits of the diagnostic information.

Strategies for sedation and anesthesia during CMR vary and often depend on institutional preference and availability of resources such as pediatric anesthesiologists with experience in CHD. Sedation in infants younger than approximately six months can be achieved by allowing them to fall into a natural sleep after a feeding [11,12]. The baby is undressed, prepared with ECG-leads and an oxygen saturation probe, and fed. As soon as the infant starts falling asleep, it is swaddled or wrapped in an immobilizer, provided noise protection, and placed on the scanner table. With this “feed, swaddle, and sleep” technique, scanning times of 30–60 minutes can be achieved. However, this approach does not allow suspension of respiration to reduce motion artifact. As the study can be compromised by early awakening, the imaging protocol should be tailored strictly to the highest priority clinical questions.

Alternatively, deep sedation can be achieved with a variety of medications (e.g., pentobarbital, propofol, fentanyl, midazolam, and inhalational agents). Meticulous care must be taken to maintain spontaneous breathing under the supervision of an experienced anesthesiology team. The principal drawbacks of this approach are an unprotected airway and reliance on spontaneous respiratory effort with the associated risks of aspiration, airway obstruction, and hypoventilation. A laryngeal mask airway may be used in conjunction with deep sedation to decrease the risk of aspiration. From an image quality standpoint, respiratory motion artifact may reduce sharpness. However, images acquired from sedated, freely-breathing patients are often still of diagnostic quality as the breathing pattern tends to be particularly consistent and thus quite amenable to commonly available respiratory motion compensation techniques.

Because of these safety and image quality concerns, some institutions prefer to perform sedation using general anesthesia with mechanical ventilation and endotracheal intubation. This approach consistently achieves adequate sedation, protects the airway, and offers control of ventilation. Respiratory motion artifact can be eliminated by suspending ventilation for brief periods (15–60 seconds) in conjunction with neuromuscular blockade. Compared to deep sedation, general anesthesia tends to require more specialized personnel and greater equipment resources (e.g., a magnetic resonance (MR) compatible anesthesia machine). Both the deep sedation and general anesthesia strategies for CMR have been shown to have a good safety profile in this fragile patient population [13-18]. MR compatible equipment should be used to monitor the heart rate, transcutaneous oxygen saturation, blood pressure, expired carbon dioxide, and body temperature. An appropriately equipped resuscitation cart and emergency management plan for the MR environment should be in place. To maximize patient safety and examination quality, it is recommended that different healthcare providers be responsible for supervising the imaging and sedation/anesthesia aspects of the study, and that both communicate closely with each other.

#### *Gadolinium-based contrast agents*

Gadolinium-based intravenous MR contrast agents (GBCA) are commonly administered in CHD patients of all ages for angiography and the assessment of myocardial perfusion and viability. These agents are often used “off-label” in children as several of them are not approved by regulatory agencies, such as the United States Food and Drug Administration or the European Medicines Agency, for pediatric-age patients. The incidence of adverse events related to GBCA in both adults and children is very low [14,19-21]. The vast majority of these reactions are mild and include coldness, warmth, or pain at the injection site; nausea; vomiting; headache; paresthesias; dizziness; and itching. Severe, life-threatening anaphylactoid or nonallergic anaphylactic reactions are quite rare (0.001% to 0.01%) [22]. There is no evidence indicating nephrotoxicity at approved dosages.

GBCA administration to patients with acute renal failure or severe chronic kidney disease is associated with the development of nephrogenic systemic fibrosis (NSF), a rare and serious condition that involves fibrosis of primarily the skin and subcutaneous tissue but may also involve the lungs, esophagus, heart, and skeletal muscles. Patients with an estimated glomerular filtration rate <30 ml/min/1.73 m^2^ are considered at highest risk. Thus, all patients who are candidates for GBCA administration should be screened for renal dysfunction, and, if identified, the most recent institutional or national guidelines regarding GBCA use should be consulted [22,23]. There are only a small number of reported cases of NSF in children (fewer than 20 as of 2010), the youngest of which was 8 years of age [24,25], and all had significant renal dysfunction [22]. No NSF cases have been reported in preterm or term neonates despite their immature kidney function and estimated glomerular filtration rates which may be < 30 ml/min/1.73 m^2^[25]. Accordingly, caution and a careful assessment of benefits and risk but not prohibition are advised when administering GBCA to neonates and infants [22,23].

#### *Scanner field strength*

Most CMR is performed on 1.5 T or 3 T scanners. In general, 3 T yields a higher signal-to-noise ratio, and therefore allows for better spatial resolution, which is particularly desirable in younger, smaller patients. This stronger signal and the inherently longer longitudinal relaxation time (T1) at 3 T, typically lead to improved clinical performance of coronary angiography, contrast-enhanced angiography, myocardial tagging, and myocardial perfusion imaging sequences. However, MR imaging at 3 T has inherently stronger off-resonance artifacts (B_0_-field inhomogeneity) and dielectric shading artifacts (B_1_-field inhomogeneity). These factors translate to significant dark band artifacts and loss of tissue contrast on steady-state free precession (SSFP) pulse sequences and signal loss on spin echo pulse sequences that are unpredictable [26]. Strategies to reduce some of these artifacts are currently under evaluation [27]. It is also worth noting that implanted metallic devices (e.g., sternal wires, stents, septal occluders, and vascular occlusion coils) are commonly encountered in CHD patients referred for CMR [28]. Device-related signal loss artifact from T2* effects is typically more pronounced at higher field strengths. Moreover, CMR compatibility information for devices is more commonly available at the 1.5 T than the 3 T field strength.

### Common CMR techniques (modules) in CHD

This section focuses only on established CMR techniques which are in routine use throughout the CMR community and are available from all scanner manufacturers. The authors readily acknowledge that newer applications such as 3D phase-contrast flow measurement, real-time and 3D cine imaging, and blood pool contrast agents can be useful when performing CMR in CHD patients.

#### *Spin echo*

**Figure 1:**
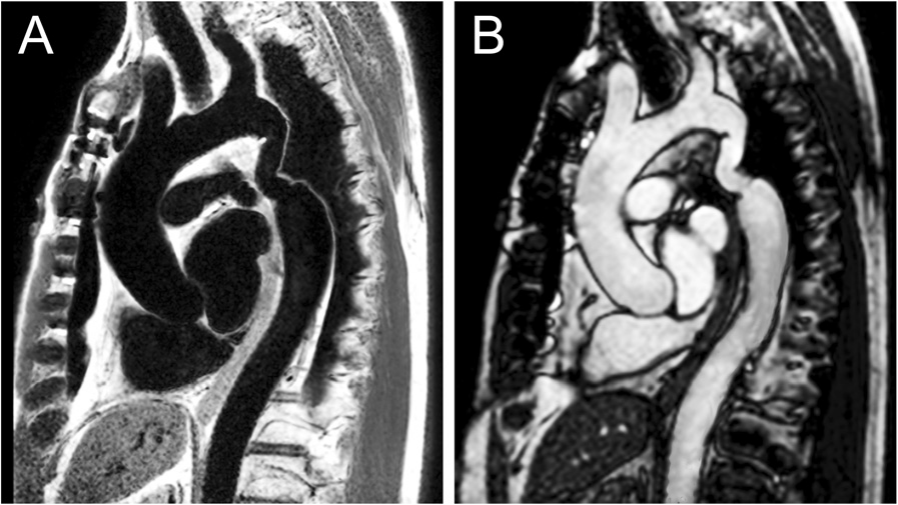
**Spin echo and gradient echo cine.** 63-year-old patient who has undergone surgical repair of coarctation of the aorta. The images are oriented parallel to the long-axis of the aortic arch and shown in diastole. **A.** ECG-triggered fast spin echo sequence acquired with a double inversion preparation pulse to suppress signal from flowing blood. Note that the resulting signal from blood is dark. **B.** ECG-gated steady-state free precession cine sequence. Note the signal from blood is bright.

Spin echo pulse sequences are typically used in CMR to generate images in which flowing blood appears dark and more stationary tissues appear as varying shades of gray or white (Figure 1). The most common variants of this technique are fast (turbo) spin echo (commercial names: TSE, Siemens; FSE, General Electric; TSE, Philips) and single-shot fast (turbo) spin echo (commercial names: HASTE, Siemens; SSFSE, General Electric; SSTSE, Philips). Both versions usually employ ECG-triggering to compensate for cardiac motion and preparation pulses to suppress signal from blood and improve image contrast. Respiratory motion can be addressed by breath-holding, acquiring multiple signal averages, triggering from respiratory bellows, or respiratory-navigator gating. By utilizing half-Fourier *k*-space filling, the single-shot technique acquires all the data to make an image in one heartbeat and is thus very time efficient. The fast spin echo technique collects data for one image over multiple heartbeats and thus takes longer than the single-shot technique, but it produces higher resolution images.

**Figure 2:**
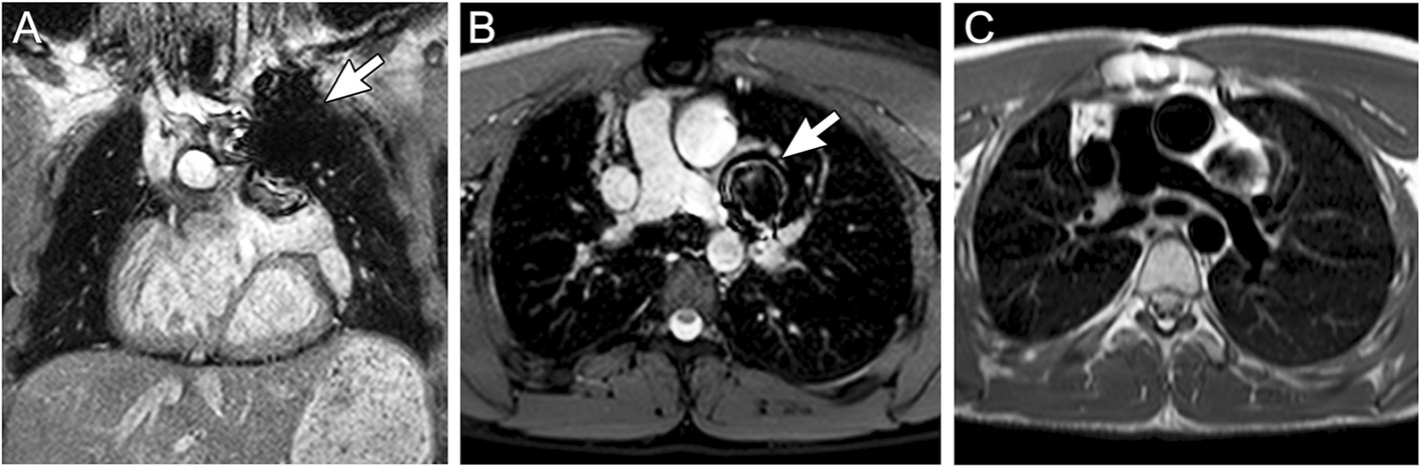
**Stainless steel coil artifact.** 17-year-old patient with dextrocardia, double-outlet right ventricle, and pulmonary stenosis who has undergone a Rastelli operation and catheter implantation of a single stainless steel vascular coil to occlude a small left superior vena cava draining to the left atrium. ECG-gated steady-state free precession sequence in a coronal plane (**A**) and axial plane (**B**) demonstrating a region of signal loss in the upper left chest (arrow) resulting from the coil. The area of signal loss is several times larger than the coil and obscures a portion of the left pulmonary artery. (**C**) shows an ECG-triggered fast spin echo sequence with a double inversion preparation pulse acquired in diastole with a similar orientation as in (**B**) With this sequence, the extent of signal loss artifact is reduced, and the left pulmonary artery is more clearly visualized.

In contrast to cine gradient echo techniques, spin echo images are usually acquired during only one portion of the cardiac cycle and do not depict motion; they are thus typically used to provide anatomic information. Their main advantage over cine imaging is that they are less susceptible to artifacts caused by turbulent flow and metallic implants such as sternal wires, septal occluders, stents, and vascular occlusion coils (Figure 2) [28]. In addition, a thinner slice thickness (≈2 mm) can be achieved which may be particularly useful in smaller patients. Fast spin echo sequences can also be modified to alter image contrast (e.g., T1- and T2-weighting, and fat suppression) and thereby help characterize tissue composition.

For smaller patients, the operator should use a smaller voxel size (adjusting both in-plane resolution and slice thickness) to ensure adequate spatial resolution (Table 1). If needed, the reduction in signal-to-noise ratio that results may be offset by employing multiple signal averages. Data acquisition should be timed to the portion of the cardiac cycle in which the heart has the least motion (i.e., the rest period) to minimize blurring. At lower heart rates the rest period is often in mid-diastole; whereas at higher heart rates (> 90-100 bpm) it may be at end-systole. Fast spin echo sequences designed for heart rates seen in adults usually acquire data every heartbeat or every second heartbeat and thus the repetition time (TR) is equal to 1 R-R or 2 R-R intervals, respectively. At higher heart rates, the R-R interval becomes shorter and the time between data acquisition will decrease resulting in less time for longitudinal signal recovery and thus lower image quality. For rates greater than 100 bpm, it may be helpful to compensate for this effect by acquiring image data every third or fourth heartbeat. Higher heart rates are also accompanied by faster cardiac motion. With the fast spin echo sequence, the number of echoes during the readout (echo train length) can be decreased to reduce the image acquisition (shot) duration and better resolve rapidly moving structures. With some implementations of spin echo imaging, the blood suppression preparation pulse may become ineffective at faster heart rates and blood will appear brighter. Finally, the default blood suppression preparation pulse will not be effective following the administration of GBCA; thus, in most protocols evaluating anatomy, spin echo sequences should be performed prior to contrast agent administration.

**Table 1:** Fast (turbo) spin echo

	**Infant/small child**		**Large child/adult**
**In-plane resolution (mm)**	1.0-2.0		1.5-2.5
**Slice thickness (mm)**	2-3		4-6
**Echo train length**	12-24		16-32
**Image acquisition timing**	3-4 R-R		1-2 R-R
**Respiratory compensation**	Free-breathing	Breath-holding	Breath-holding
**Number of signal averages**	3	1-2	1
**Trigger delay**	Diastole or systole		Diastole

#### *Gradient echo cine*

Gradient echo cine pulse sequences generate images in which flowing blood appears bright (Figure 1). With the use of ECG-gating, it produces multiple images across the cardiac cycle that can be displayed in cine loop format to visualize motion, one of its main advantages over spin echo sequences. Respiratory motion artifact can be minimized by breath-holding (preferred when possible) or by acquiring 2–4 signal averages with the patient breathing. In clinical practice, this imaging sequence is often prescribed across the anatomy of interest to yield a stack of contiguous cross-sectional slices that can be displayed in a multi-location, multi-phase format.

Gradient echo cine imaging can be performed using a standard spoiled gradient echo pulse sequence or the subsequently developed SSFP sequence (commercial names: TrueFISP, Siemens; FIESTA, General Electric; balanced-FFE, Philips). SSFP imaging is faster and provides superior contrast between blood and myocardium compared to standard gradient echo imaging and is thus more commonly used. The SSFP sequence is also relatively less sensitive to flow disturbances caused by stenotic or regurgitant jets. However, it is also more prone to image artifact when there are inhomogeneities in the magnetic field (B_0_) caused by suboptimal scanner shimming or implanted ferromagnetic devices. Thus, the standard gradient echo technique may be preferred for visualization of flow jets or imaging near implanted devices such as stents and mechanical valves.

When imaging smaller structures in younger patients, the spatial resolution should be increased appropriately (Table 2). Changes to accomplish this (e.g., increased matrix size, smaller field of view, thinner slice thickness), however, will prolong the echo time (TE). Once the TE becomes > 2 ms and the TR > 4 ms, the quality of SSFP imaging often deteriorates. One can increase the matrix size only in the phase encode direction to improve resolution without prolonging the TE, but this is done at the expense of increased acquisition time. Thus, a careful balance must be struck between spatial resolution, acquisition time, and image quality. Alternatively, some centers prefer to use high-resolution standard gradient echo imaging with free-breathing and multiple signal averages.

**Table 2:** Cine steady-state free precession

	**Infant/small child**		**Large child/adult**
**In-plane resolution (mm)**	1.2-2.0		1.5-2.5
**Slice thickness (mm)**	4-6		5-8
**Inter slice gap (mm)**	0-2		0-4
**Respiratory compensation**	Free-breathing	Breath-holding	Breath-holding
**Number of signal averages**	3	1	1
**Reconstructed phases per R-R interval**	20-30		
**ECG gating**	Retrospective		

#### *Contrast-enhanced magnetic resonance angiography*

**Figure 3:**
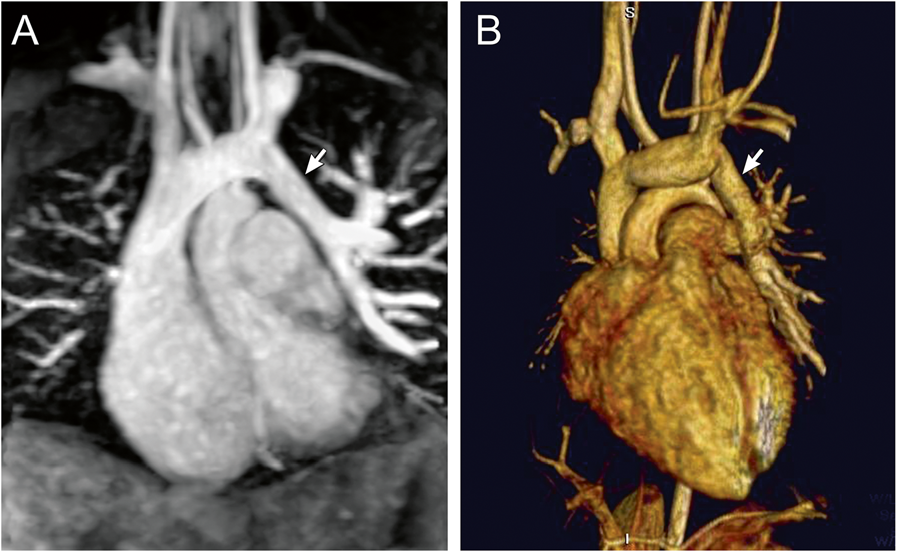
**Contrast-enhanced magnetic resonance angiography.** 9-year-old patient with partially anomalous pulmonary venous return of the left upper pulmonary vein (arrow) to the leftward aspect of the left innominate vein. Contrast-enhanced magnetic resonance angiogram shown in a coronal plane using a sub-volume maximal intensity projection (**A**) and volume rendering (**B**).

Magnetic resonance angiography (MRA) using an intravenously administered GBCA can produce a high-resolution, high-contrast three-dimensional (3D) dataset of the entire chest vasculature in a short scan time, typically less than 30 seconds (Figure 3). As CHD is frequently associated with abnormalities of the chest vasculature, this technique is often employed in both pre- and post-operative CMR imaging protocols. Studies demonstrating its utility and accuracy in patients with CHD have been published for examination of the aorta and its branches, pulmonary arteries, pulmonary veins, systemic veins, aortopulmonary and venous-venous collateral vessels, systemic-to-pulmonary artery shunts, conduits, and vascular grafts [29-34]. The 3D datasets generated by MRA are well-suited to volume rendered displays which can enhance understanding of complex spatial relationships and are more comprehensible to non-CMR specialists (Figure 3). Nevertheless, it is essential that the source data be carefully reviewed by the reporting physician as anatomic information may be omitted or distorted by the volume rendering algorithm.

When possible, MRA should be performed with breath-holding to minimize artifact from respiratory motion [35]. In smaller patients, one should ensure that spatial resolution, both in-plane and partition thickness, is sufficient. A contrast agent dose of 0.1-0.2 mmol/kg is typically used. The time delay between the start of contrast injection and data acquisition will influence which portions of the vasculature will be depicted. This interval can be determined by a MR fluoroscopic method that allows real-time visualization of the arrival of the contrast bolus to the targeted region, by a preceding timing run with a smaller dose of contrast, or by automatic bolus detection. Because the effect of the contrast agent persists even during recirculation, two or three sequential MRA data acquisitions are useful to ensure visualization of all the vasculature. More recently, imaging acceleration techniques have been applied to shorten the acquisition time to 2–5 seconds thereby permitting multiple 3D volume sets to be acquired as the contrast agent passes through the circulation producing a “time-resolved” MRA [36-39]. A smaller contrast dose (0.05-0.1 mmol/kg) is often used. This time-resolved approach has several potential advantages: 1) observing the passage of contrast may have diagnostic benefits, 2) timing of the acquisition is less critical because multiple volume sets are obtained as the contrast passes through the circulation, and 3) it may have less sensitivity to respiratory motion artifact. The principal disadvantage with this technique is that the decrease in acquisition time is usually achieved in part from a reduction in spatial resolution which may hinder diagnostic accuracy, particularly in smaller patients. In addition, the undersampling of *k*-space used to accelerate the acquisition may result in image artifact.

Both the standard and time-resolved contrast MRA techniques do not utilize ECG-gating; therefore, motion over the cardiac cycle causes blurring, particularly of the aortic root, coronary arteries, and intracardiac structures. Another disadvantage is that the use of GBCA incurs some risk of adverse reactions (see above).

#### *ECG and respiratory navigator-gated 3D SSFP*

**Figure 4:**
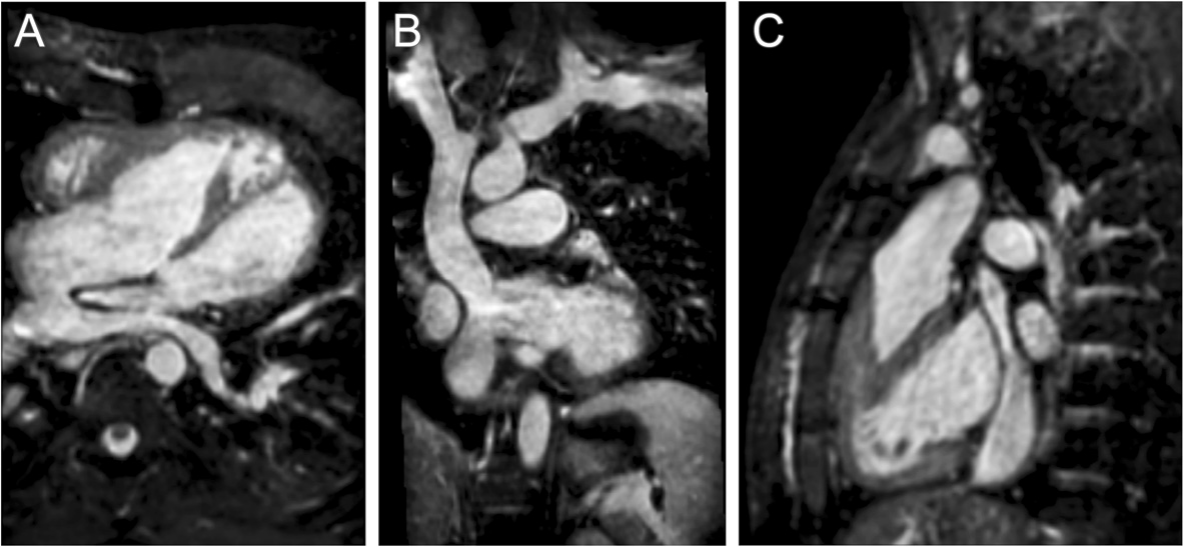
**3D steady-state free precession.** A patient with transposition of the great arteries who has undergone a Senning operation. An ECG and respiratory navigator-gated 3D SSFP sequence was utilized to generate a 3D volume with 1.5 mm isotropic resolution timed to mid-diastole. The navigator efficiency was 45% and the acquisition time was 6 minutes. Multiplanar reformatting of this volume allows a comprehensive morphologic evaluation of the heart and great vessels including the Senning pathways (**A**, **B**, and **C**).

In its typical implementation, the ECG and respiratory navigator-gated 3D SSFP technique delivers a 3D anatomic dataset with an isotropic voxel size of approximately 1.2-2.0 mm without the use of a contrast agent (Table 3). Its utility and validation have been reported in patients with CHD [40-42]. Using ECG triggering, the data acquisition is confined to one or two portions of the cardiac cycle thereby minimizing blurring from cardiac motion. Intracardiac anatomy and coronary arteries can thus be more clearly visualized than with contrast-enhanced MRA. The 3D SSFP sequence is performed with free-breathing. Respiratory motion is compensated for by gating data acquisition to expiration with the use of a navigator beam tracking the motion of the diaphragm. This approach allows for improvement in spatial resolution including isotropic voxel size because scan time is not limited to the duration of a single breath-hold. The isotropic property of the anatomic data permits arbitrary reformatting in any desired imaging plane during review without the loss of resolution (Figure 4).

**Table 3:** ECG and respiratory navigator-gated 3D steady-state free precession

	**Infant/small child**	**Large child/adult**
**Isotropic resolution (mm**^**3**^**)**	1.2-1.5	1.3-2.0
**Navigator window (mm)**	3	5
**Image acquisition duration (ms)**	40-60	80-150
**Trigger delay**	End-systole or mid-diastole	Mid-diastole

The 3D SSFP sequence has four principal disadvantages. First, its acquisition time is relatively long, usually approximately 7–10 min, during which the patient must be absolutely still. Younger children may have difficulty with this level of cooperation. Second, the sequence is very susceptible to artifacts caused by turbulent flow and magnetic field inhomogeneity such as that caused by presence of stents or other ferromagnetic implants. Therefore, vessels with stenosis or regurgitation, and structures around stents can sometimes be misinterpreted. Third, the data is confined to one or two portions of the cardiac cycle thereby precluding the evaluation of cardiac and vessel motion. Finally, as with other ECG-gated techniques, image quality will suffer when the patient has an irregular heart rhythm. In such cases, generating stacks of thin SSFP localizer type images in the cardinal planes during a breath-hold or free-breathing with 2–3 signal averages may be a useful alternative.

**Figure 5:**
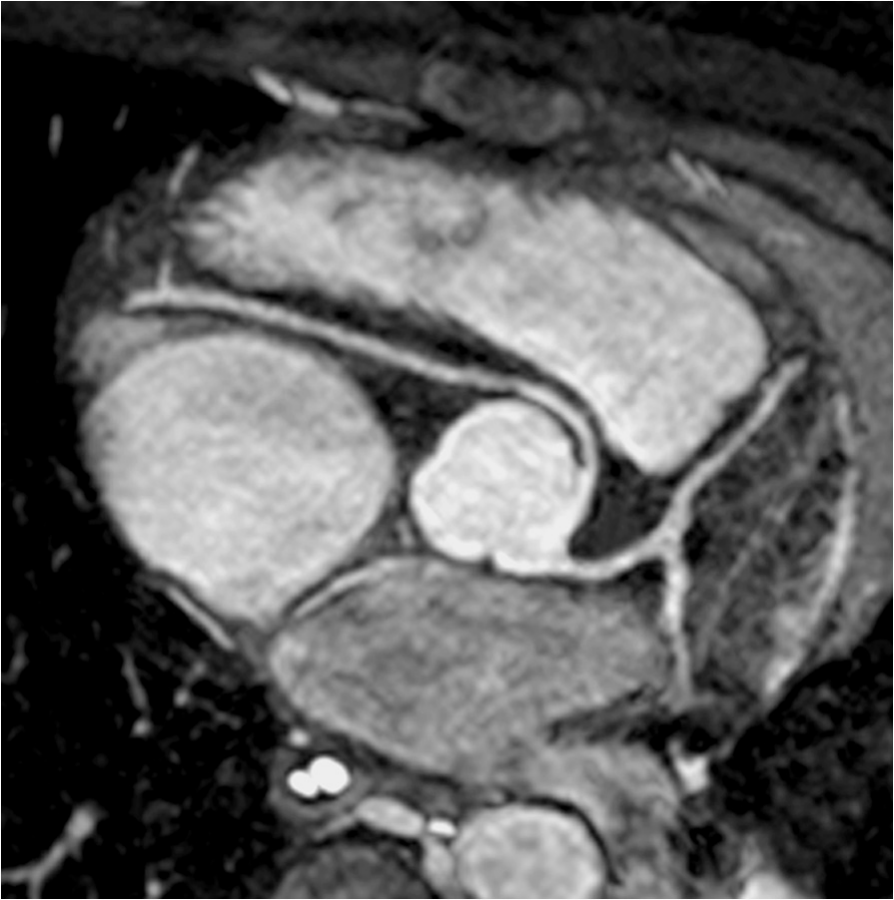
**Coronary MR angiography.** Patient with anomalous origin of the right coronary artery from the left aortic sinus of Valsalva. Coronary angiography was performed using an ECG and respiratory navigator-gated 3D SSFP sequence with data acquisition timed to the diastolic rest period of the cardiac cycle. Multiplanar reformatting oriented in short-axis to the aortic root yielded this image.

A version of 3D SSFP is generally the technique of choice for coronary artery imaging in patients with CHD (Figure 5) [43-46]. For this purpose in particular, data acquisition must be confined to the rest period of the cardiac cycle (i.e., that with the least motion) to minimize blurring of these small, fast moving structures. The rest period of the heart is chosen by reviewing a high temporal resolution cine image of the heart (≥50 images per cardiac cycle), usually a 4-chamber view, and identifying the appropriate trigger delay and acquisition (shot) duration. Younger patients will typically have faster heart rates and thus require a shorter acquisition duration. Moreover, at higher heart rates (> 90-100 bpm) the optimal rest period may be in end-systole. If a patient has difficulty being still during the scan and the primary diagnostic goal is to visualize the proximal coronary arteries (e.g., suspect anomalous origin of a coronary artery), it may be helpful to shorten the scan duration by prescribing a smaller, more targeted imaging volume around the aortic root rather than the entire heart.

#### *Ventriculography*

CMR ventriculography generates cine images of the ventricles that enable calculation of ventricular volume, mass, and ejection fraction as well as evaluation of regional wall motion. It is a specialized application of gradient echo cine imaging described above. CMR is widely considered the clinical reference standard for ventriculography. It is particularly useful in situations where echocardiography has significant limitations such as in patients with poor acoustic windows and the evaluation of the right ventricle or single ventricle. It is a key component of the CMR functional evaluation in patients with CHD.

In addition to the technical points mentioned in the gradient echo cine imaging section, the use of retrospective rather than prospective ECG-gating is important so that the entire diastolic portion of the cardiac cycle is evaluated. When possible, breath-hold acquisitions are recommended, preferably at end-expiration as diaphragmatic position tends to be more consistent from breath-hold to breath-hold [47,48]. Parallel imaging or partial Fourier techniques can be utilized to decrease breath-hold time but at the expense of image quality (e.g., poorer signal-to-noise ratio). Alternatively, free-breathing imaging with the patient instructed to breathe regularly and quietly, and multiple signal averaging (2–4) can be performed. Proper attention to prescribing an appropriate temporal resolution is essential to adequately depict cardiac motion and capture the end-systolic phase of the cardiac cycle. Cine CMR sequences often employ view sharing (also called echo sharing) to increase the apparent temporal resolution by undersampling *k*-space data for certain frames and utilizing data from adjacent frames to fill the missing data [49]. Although these interpolated frames smooth cardiac motion, they do not increase the true temporal resolution of the sequence which can be calculated as the product of the TR and lines per segment (also called views per segment or turbo field echo (TFE) factor). It is recommended that a minimum of 15 non-interpolated images be acquired over the cardiac cycle (R-R interval/(TR•lines per segment) ≥ 15). At the higher heart rates typically seen in younger children, this will require that the number of lines per segment be decreased to maintain adequate temporal resolution.

**Figure 6:**
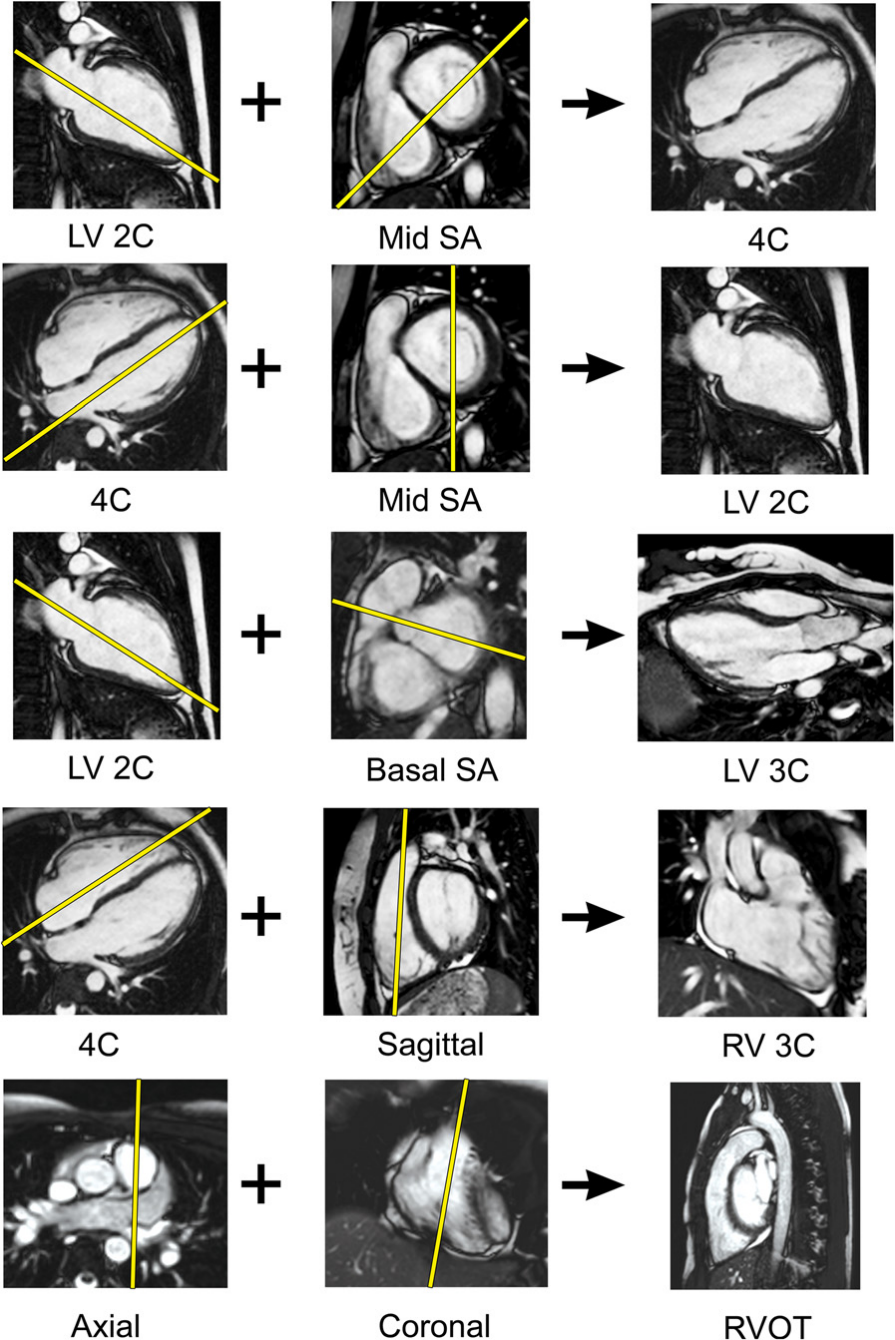
**Ventricular views.** The diagram illustrates one approach to planning standard ventricular views (right column) based on adjusting the slice locationon two other views (left and middle columns). Note that for the 4C, LV 2C, and LV 3C, the imaging plane is carefully positioned to pass through the apex of the LV and bisect the mitral valve plane. LV, left ventricle; RV, right ventricle; RVOT, right ventricular outflow tract; SA, short-axis; 2C, 2-chamber; 3C, 3-chamber; 4C, 4-chamber.

**Figure 7:**
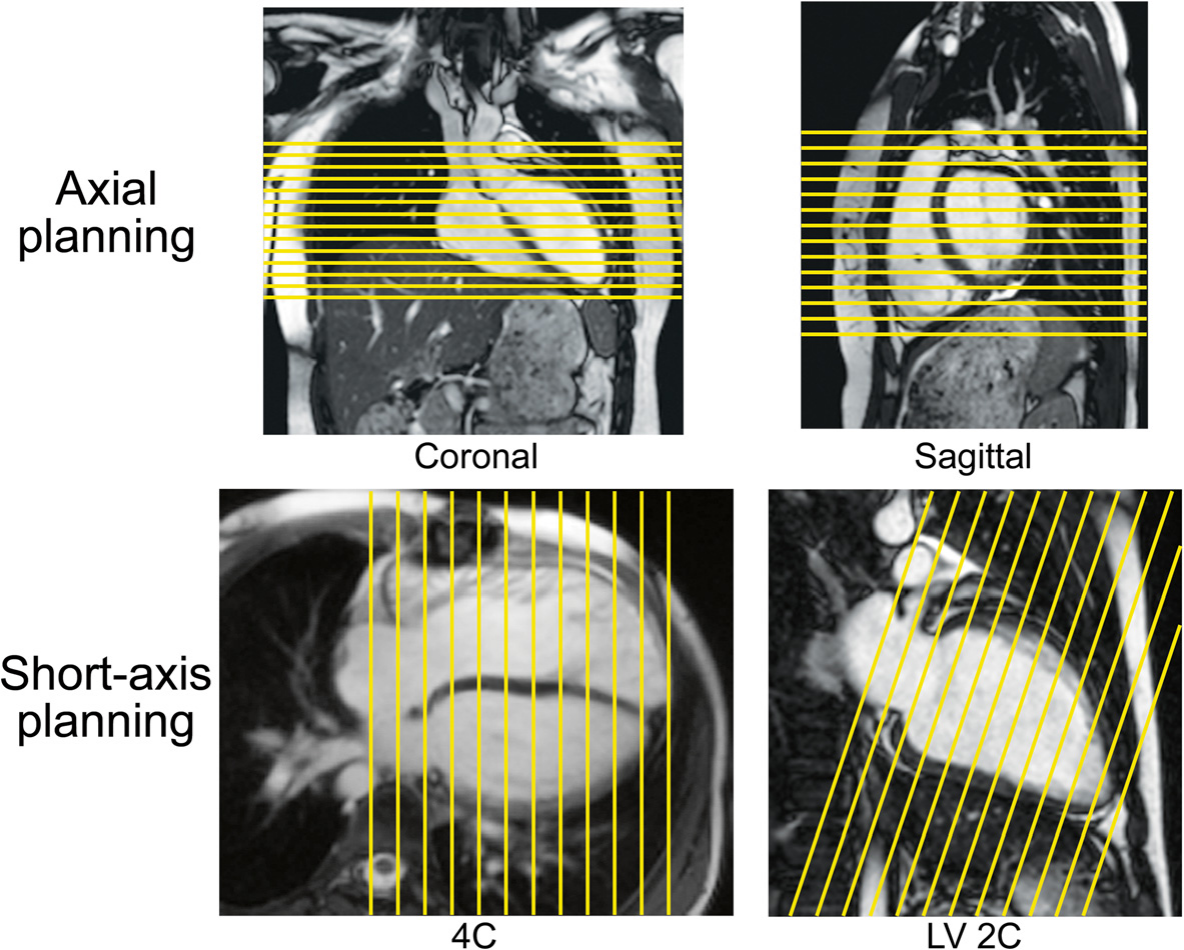
**Planning for ventriculography.** An axial stack of cine images for ventriculography is planned by adjusting the slice locations on both coronal and sagittal images (top row). A short-axis stack of cine images for ventriculography is planned by adjusting the slice locations on 4-chamber (4C) and left ventricular 2-chamber (LV 2C) images in diastole (bottom row). Note that both the axial and short-axis stacks are prescribed to ensure complete coverage of the left and right ventricles. In this short-axis example, the slices are oriented perpendicular to the ventricular septum on the 4C view, and care is taken to ensure that coverage includes the anterior portion of the dilated right ventricle which extends above the tricuspid valve plane. An alternative short-axis planning approach is to orient the slices parallel to the atrioventricular valve plane on the 4C view (not shown).

Imaging planes for ventriculography should be planned carefully and include the following (Figure 6): 1) 4-chamber (horizontal long-axis) view, 2) left ventricular 2-chamber (vertical long-axis) view, 3) left ventricular 3-chamber view including the mitral valve inflow and left ventricular outflow tracts, 4) right ventricular 3-chamber (right anterior oblique) view including tricuspid valve inflow and right ventricular outflow, and 5) a stack of contiguous slices which completely encompass both ventricles (Figure 7). The data from the ventricular stack are used to calculate ventricular end-diastolic volume, end-systolic volume, stroke volume, ejection fraction, and myocardial mass using the summation of disks method. If the patient is expected to have limited tolerance for the examination, acquiring the ventricular stack should be prioritized.

There is practice variation regarding the orientation of the ventricular stack. Some centers prefer to prescribe it in a ventricular short-axis plane parallel to the atrioventricular valve plane or perpendicular to the ventricular septum (assisted by cross-referencing with the long-axis images), and others prefer to prescribe an axial plane (Figure 7) [50-55]. Furthermore, some advocate acquiring data sets in both the short-axis and axial planes, or two sets of short-axis planes—one oriented parallel to the mitral valve plane and one oriented parallel to the tricuspid valve plane as these may be offset, particularly when there is right ventricular dilation. The principal advantages of the axial plane over the short-axis plane include 1) straightforward planning especially in patients with complex ventricular morphology, 2) easier identification of the atrioventricular boundary during post-processing, and 3) more anatomic coverage of non-ventricular structures (e.g., atria). Among its disadvantages are 1) difficulty in evaluating left ventricular segmental wall motion using established guidelines, and 2) a limited evaluation of ventricular mass because the epicardial and endocardial borders of the diaphragmatic wall of the heart are not clearly defined. With either approach, it is essential that the entire ventricle be included in the image stack. For the short-axis orientation, cross-referencing the slices on the 4-chamber view may reveal the need to extend the stack above the tricuspid valve plane in patients with a dilated right ventricle (Figure 7).

**Figure 8:**
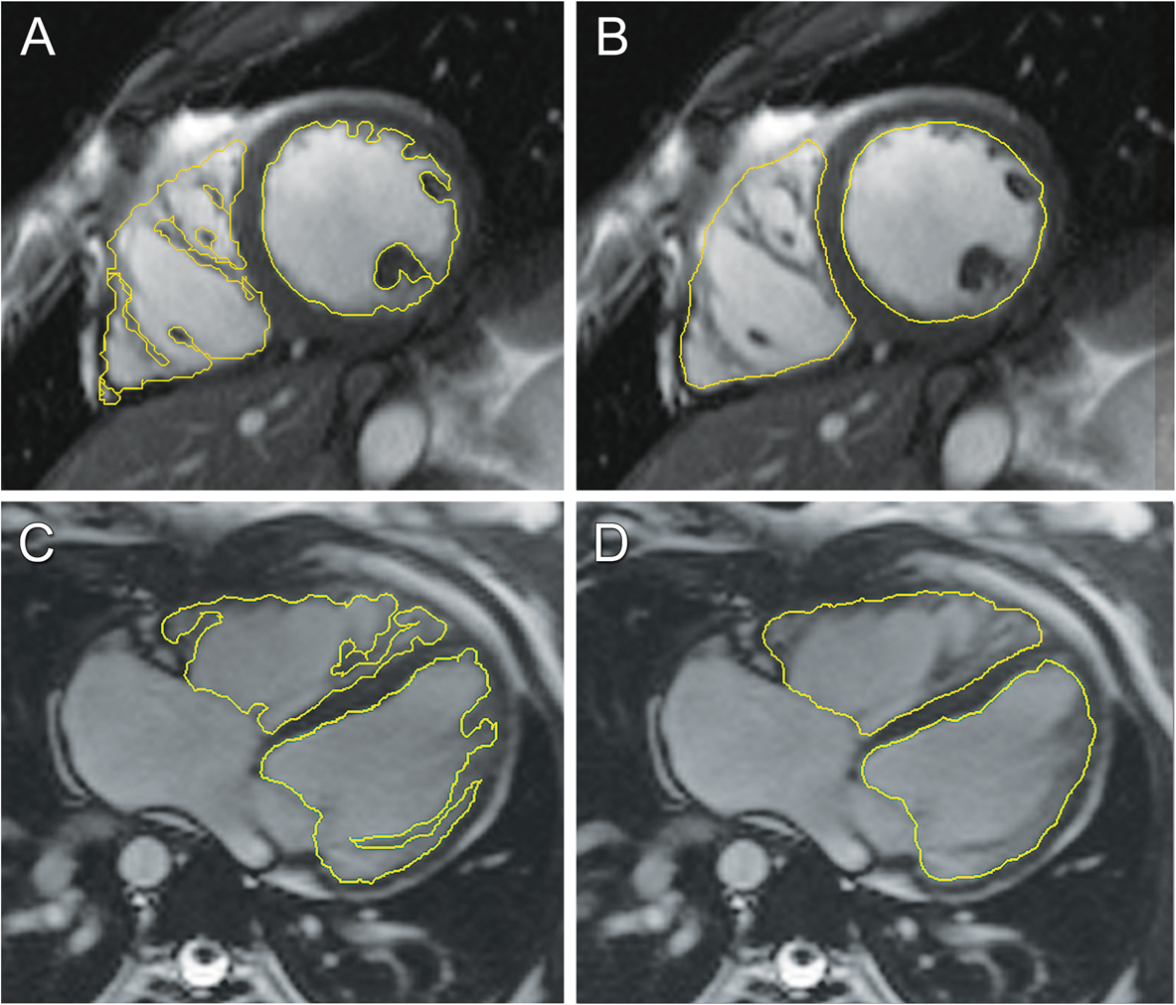
**Tracing ventricular borders.** Diagram demonstrating the drawing of left and right ventricular endocardial contours in end-diastole. Images may be acquired in a ventricular short-axis orientation (**A** and **B**) or in an axial orientation (**C** and **D**). There is practice variation regarding whether to trace the papillary muscles and right ventricular trabeculations to exclude them from (**A** and **C**) versus include them in (**B** and **D**) the blood pool.

The ventricular image stack is typically analyzed by demarcating endocardial and epicardial borders with the assistance of software tools (Figure 8). Cross-referencing the ventricular short-axis images with long-axis images and observing wall motion in a cine fashion facilitate accurate determination of the atrioventricular and semilunar valves planes [56,57]. In the absence of right ventricular hypertrophy, the epicardial boundary of the thin-walled right ventricle can be difficult to detect. When the left ventricle is in its normal systemic position, the mass of the ventricular septum is counted as part of the total left ventricular mass. When the right ventricle is in the systemic position, there is no consensus regarding to which ventricle the septal mass should be allocated. In patients with ventricular conduction delay, which is relatively common in CHD, the end-systolic and end-diastolic frames (i.e., time point in the cardiac cycle) may not be the same for the right and left ventricles and should thus be selected independently to yield the minimum and maximum volumes respectively. There is practice variation regarding whether to trace the papillary muscles and right ventricular trabeculations in order to exclude them from blood pool and count them toward ventricular mass (Figure 8) [58-61]. Tracing these structures will yield smaller ventricular volumes, with little change in stroke volume but a higher ejection fraction. Though theoretically more accurate, this approach is time consuming in the absence of a reliable automated system and may reduce measurement reproducibility.

Normative data on ventricular parameters by CMR are available for both adults and older children [50,52,53,62,63]; there is still a need for robust data in patients younger than 8 years. Given the practice variations in the methods of acquiring and analyzing the ventriculography image data noted above, it is recommended that a center’s approach to these issues correspond to that used in the normative dataset that has been selected as the center’s reference values. Although indexing ventricular volumes to body surface area is a common practice, this does not fully account for changes in body size from infancy to adulthood as the volumes do not vary linearly with body surface area [52,64,65]. Thus, a given indexed volume (e.g., end-diastolic volume of 80 ml/m^2^) may be normal for an adult but above normal for an infant.

One of the strengths of CMR ventriculography is that studies have demonstrated very good reproducibility in children and adults with CHD [58,66-69]. To achieve this level of quality and reliability, centers should maintain a rigorous and consistent approach to imaging and analysis. Furthermore, to optimize interstudy reproducibility in patients followed longitudinally, the ventricular borders demarcated in the analysis software should be saved so that they can be compared side-by-side with those from subsequent studies.

#### *Blood velocity and flow measurement*

Velocity-encoded phase-contrast (PC) cine CMR is the primary technique used to measure blood flow velocity and volume. A detailed description can be found elsewhere [70-73]. In brief, PC CMR is based on the principle that the signal from hydrogen nuclei (such as those in blood) flowing through specially designed magnetic field gradients accumulates a predictable and measurable phase shift that is proportional to its velocity. A PC CMR pulse sequence produces two sets of cine images: *magnitude images* that provide anatomic information and *phase images* in which the velocity information is encoded. On the phase images, the signal amplitude (brightness) of each voxel is proportional to mean flow velocity within that voxel. Maximum velocity in one direction is displayed as the brightest white, maximum velocity in the opposite direction as the darkest black, and zero velocity as mid-grey. Using specialized software, regions of interest around a vessel are defined, and the flow rate is automatically calculated from the product of the mean velocity and the cross-sectional area.

Blood flow measurements are an important element of the CMR examination of CHD patients and have variety of applications. Examples include measurement of cardiac output [74,75], pulmonary-to-systemic flow ratio (Q_p_/Q_s_) [76-81], differential lung perfusion [82-85], valvular regurgitation [55,86-94], aortopulmonary collateral flow [95-97], and pressure gradient [98-101].

For PC CMR flow measurements, the use of retrospective rather than prospective ECG-gating is preferred so that the entire diastolic portion of the cardiac cycle is evaluated (Table 4). The quality of the ECG-gating should be carefully monitored, particularly during acquisitions lasting several minutes. If the heart rate changes significantly or there are frequent invalid triggers, the sequence should be stopped and repeated. Measurements can be performed with free-breathing and multiple signal averages (2–4) or by shortening the scan time so that it can comfortably be performed within a breath-hold. The reduced scan time is usually achieved by reductions in temporal and spatial resolution, as well as faster gradient switching–all of which may increase measurement error. Moreover, physiological changes can occur with breath-holding that alter the measurements [102-104] and may confound clinical interpretation. For these reasons, the authors recommend free-breathing acquisitions. The scan time of these measurements may be reduced with the use of parallel imaging [80,105,106].

**Table 4:** Velocity-encoded phase-contrast cine for quantitative flow measurements

	**Infant/small child**	**Large child/adult**
**In-plane resolution (mm)**	1.0-1.3	1.3-2.0
**Slice thickness (mm)**	5	6-8
**Number of signal averages**	3	
**Reconstructed phases per R-R interval**	25-30	
**Velocity encoding (cm/s)**	Artery 200, vein 100, atrioventricular-valve inflow 150	
**Cardiac/respiratory motion**	Retrospective ECG-gating with free-breathing	

**Figure 9:**
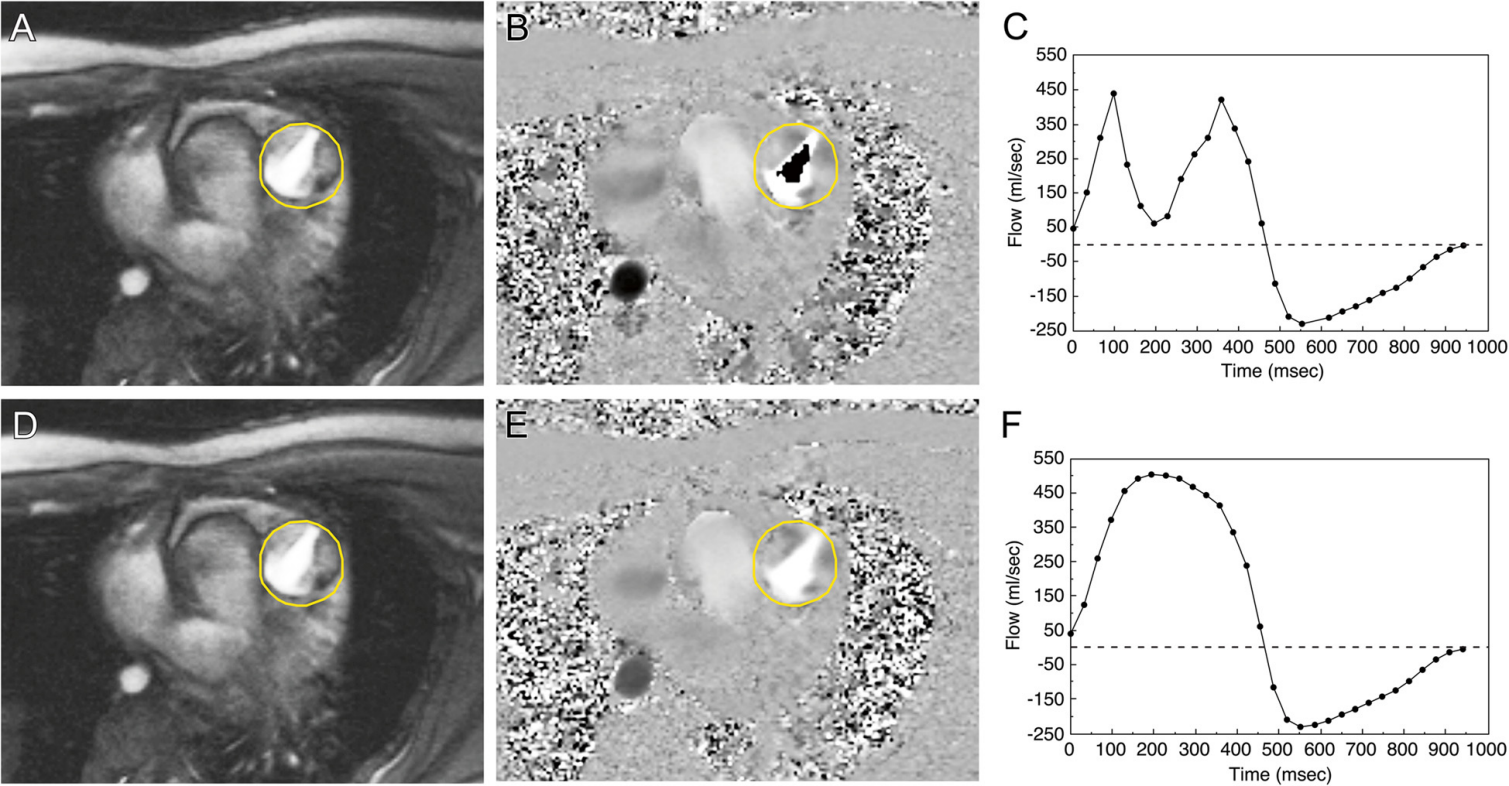
**Effect of aliasing on phase-contrast cine CMR (PC CMR) flow measurements.** Sixteen-year-old patient with surgically repaired tetralogy of Fallot and mild pulmonary valve stenosis. PC CMR was performed in the main pulmonary artery with the velocity range (v_enc_) set incorrectly at 200 cm/sec (top row) and then with the v_enc_ set correctly at 300 cm/sec (bottom row). Magnitude (**A**, **D**) and phase images (**B**, **E**) in systole, and the resulting flow curves (**C**, **F**) generated from analyzing the region of interest (yellow contour) are shown. Because the peak velocity is 260 cm/sec, aliasing (**B**) and flow underestimation (**C**) are seen with a v_enc_ of 200 cm/sec but not with a v_enc_ of 300 cm/sec (**E** and **F**).

Accurate PC CMR measurements require sufficient spatial resolution to avoid significant partial volume effects. Specifically, there should be more than 3 pixels across the diameter or more than 8 pixels in the cross-section of the vessel or cardiac valve of interest [107,108]. Proper attention to prescribing an appropriate temporal resolution is also essential because under-sampling will smooth a pulsatile flow curve and cause inaccuracies. For PC CMR, true temporal resolution (as opposed to interpolated temporal resolution) equals 2•TR•lines per segment (lines per segment is also called views per segment or TFE factor). It is recommended that a minimum of 20 non-interpolated images be acquired over the cardiac cycle (R-R interval/(2•TR•lines per segment) ≥ 20). At the higher heart rates typically seen in younger children, this will require that the number of lines per segment be decreased to maintain adequate temporal resolution. Finally, with PC CMR measurements, the operator must set the velocity range (v_enc_) prior to running the pulse sequence; velocities which exceed this range will alias and be misrepresented (Figure 9). It is recommended that the v_enc_ be set to approximately 25% above the expected maximum velocity so as to optimize the dynamic range. When blood velocity is increased, such as in the setting of valve or vessel stenosis, the operator’s choice of v_enc_ may be guided by information from recent echocardiography. If aliasing occurs, the measurement should be repeated using a higher v_enc_ or the data may be rescaled with cautious use of post-processing software.

**Figure 10:**
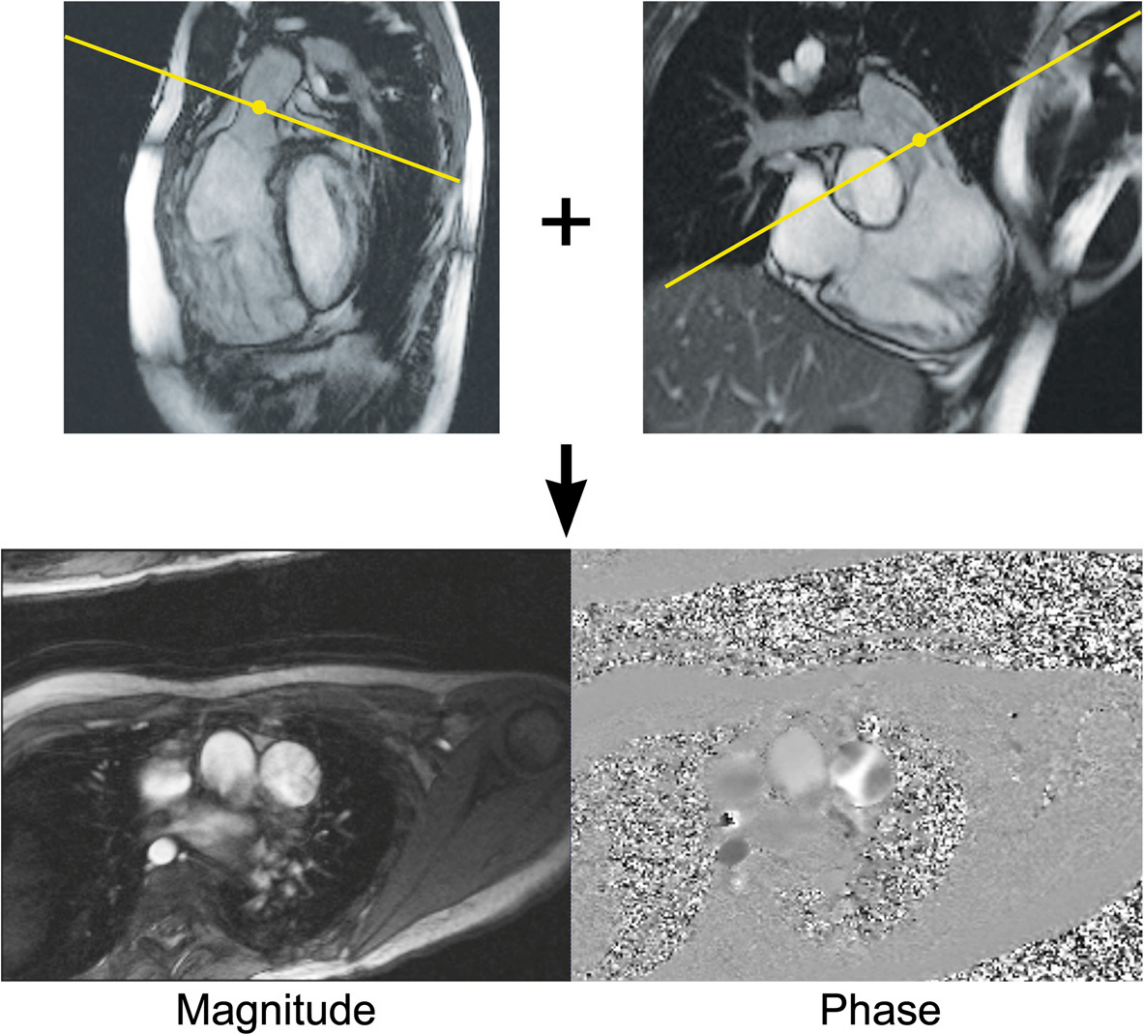
**Planning a CMR phase-contrast acquisition to measure flow in the main pulmonary artery.** The PC CMR imaging plane is simultaneously viewed and adjusted on orthogonal views of the main pulmonary artery (top row) to ensure that it is oriented perpendicular to the blood vessel. The resulting magnitude and phase images are shown (bottom row).

**Figure 11:**
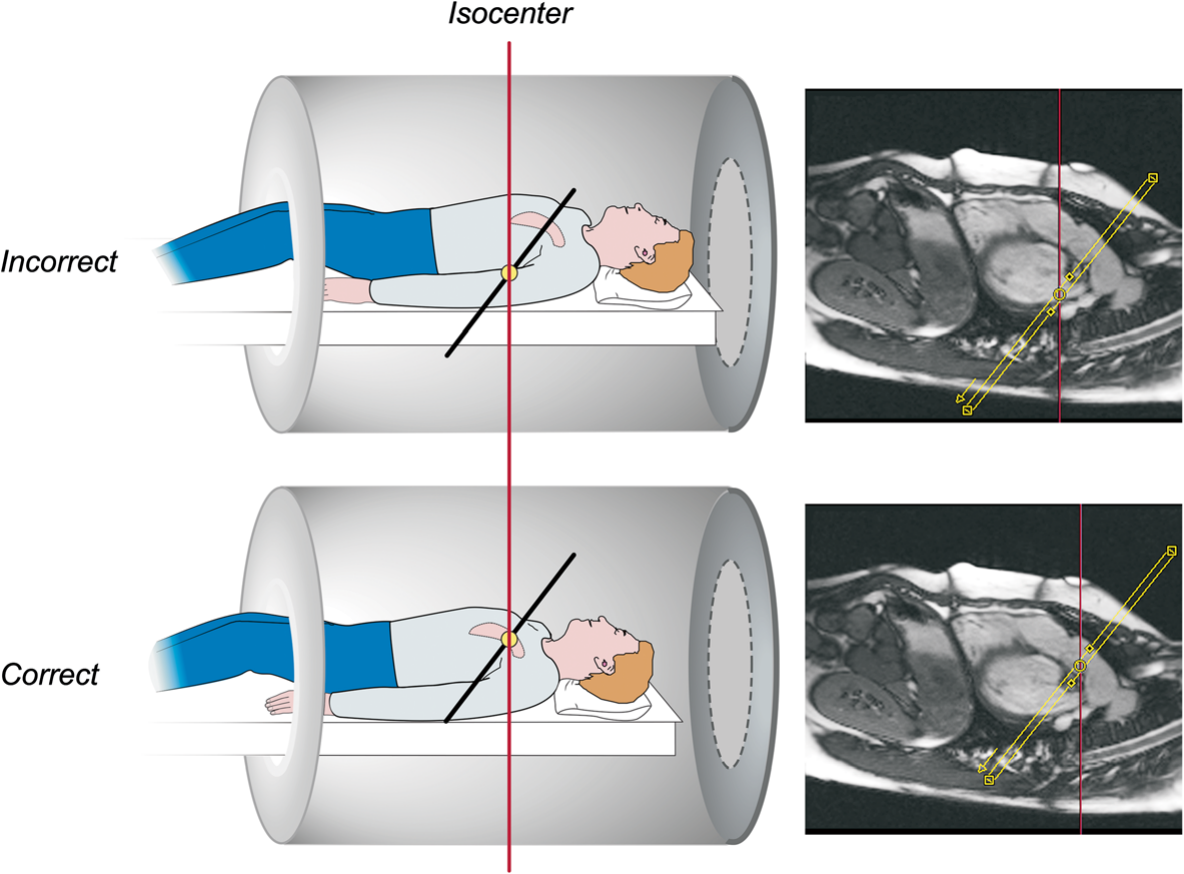
**Proper positioning of the imaging plane for main pulmonary artery blood flow measurement.** PC CMR velocity and flow measurements are most accurate when the location of interest is at the isocenter of the scanner during the acquisition. Most MR scanners will slide the patient table so that the center of the imaging plane (yellow circle) is at the scanner’s isocenter (vertical red line). Prescribing the imaging plane so that the center of the image is at the same level in the superoinferior dimension as the location of interest is therefore recommended.

Careful positioning of the PC CMR imaging plane is essential for accurate velocity and flow measurements. It should be aligned strictly perpendicular to the blood vessel’s or valve’s orientation using two orthogonal planning views in order to minimize partial volume effects (Figure 10), and the velocity encoding direction should be set to the through-plane direction. The vessel of interest should be as close to the scanner’s isocenter as possible to maximize gradient fidelity (Figure 11). This is accomplished by prescribing the plane so that the center of the image is at the same level as the vessel in the superoinferior direction. The scanner will then slide the patient table so that the center of the imaging plane and thus the vessel is close to the scanner’s isocenter. Placement of the imaging plane in regions of turbulent flow should be avoided as such flow can lead to signal loss and inaccuracies. Similarly, positioning of the imaging plane away from ferromagnetic implanted devices is recommended as these disrupt the magnetic field gradients.

There is some uncertainty and practice variation regarding the optimal location for semilunar valve flow measurement. A position closer to the annulus has the potential advantages of a less complex flow pattern, reduced impact of vessel compliance, and exclusion of coronary flow in the case of the aortic valve [109]. Through-plane motion, however, is greatest near the annulus, and this contributes to measurement error [110,111] and precludes precise placement between the annulus and coronary ostia throughout the cardiac cycle using standard two-dimensional (2D) PC CMR. Most operators typically place the imaging plane at the sinotubular junction or proximal ascending aorta to measure aortic valve flow, and in the middle of the main pulmonary artery to measure pulmonary valve flow [112]. Atrioventricular valve inflow or regurgitation volume can be quantified indirectly through several different comparisons of ventricular stroke volume measured by ventriculography and semilunar valve flow measurement by PC CMR [55,88,94,112-115]. Atrioventricular valve inflow can also be quantified directly with PC CMR by prescribing a location perpendicular to the inflow direction [116,117]; to ensure that this plane remains apical to the valve throughout the cardiac cycle, it should be positioned at the annular level on an end-systolic image. Again, through-plane motion will compromise the accuracy of flow measurements made with conventional 2D PC CMR [111,118,119].

PC CMR has been utilized to estimate the pressure drop across discrete stenoses in valves and vessels by measuring the maximum flow velocity and applying the modified Bernoulli equation. A useful way to plan this is to prescribe a PC CMR imaging plane parallel to the direction of blood flow setting the velocity encoding direction in the same in-plane direction, and then to prescribe a second PC CMR acquisition with through-plane encoding perpendicular to the location of the peak in-plane velocity. However, the authors recommend that PC CMR measurements of peak velocity be applied cautiously as there are a number of factors that may lead to erroneous velocity measurements (typically an underestimate) including difficulty aligning with complex flow jets, partial volume effects, insufficient temporal resolution, signal loss, and misregistration artifacts.

As with PC CMR acquisitions, the post-processing of the image data requires careful attention to detail. The target vessel must be accurately identified and a region of interest should be drawn on the outer margin of the lumen. Automatic border detection tools in most software programs are fairly accurate; however, the contours should be reviewed on each image, as the vessel moves and changes size during the cardiac cycle. Abnormal signal, such as susceptibility artifact from air in the lungs, should be carefully excluded from the region of interest.

PC CMR velocity and flow measurement, like all quantitative techniques in clinical medicine, has sources of error and limitations, and it is essential that reporting physicians have a good understanding of them. These include inappropriate setting of the v_enc_, signal loss with complex turbulent flow, partial volume averaging, signal misregistration, and phase offset errors due to eddy currents or uncorrected concomitant gradients [70-72,110,120,121]. Adherence to the guidelines above will minimize these concerns but phase offset errors are particularly troublesome as they are often difficult to detect and may have a significant impact on accuracy. On some scanners it may be advisable to subtract the background velocity or flow measured on a stationary phantom from that measured on the patient’s PC CMR images [122-124]. Through-plane motion of the heart and blood vessels may impact the accuracy of flow measurements [110,111,118]. 3D PC CMR sequences along with specialized post-processing analysis software can compensate for this motion though its use is not yet widespread [119,125-129]. In all cases, PC CMR data should be scrutinized to ensure that they are consistent with the known information about the patient’s condition and with the other CMR data from the examination. For instance, the net flow in the main pulmonary artery and ascending aorta should be approximately equal in patients without any evidence of a shunt, net main pulmonary artery flow should equal the sum of the net branch pulmonary artery flow, and flow in a great vessel should agree well with the corresponding ventricular stroke volume from ventriculography (in the absence of atrioventricular valve regurgitation or a shunt). When data is incongruous, one of the known limitations described above can usually be identified.

#### *Vasodilator myocardial perfusion*

**Figure 12:**
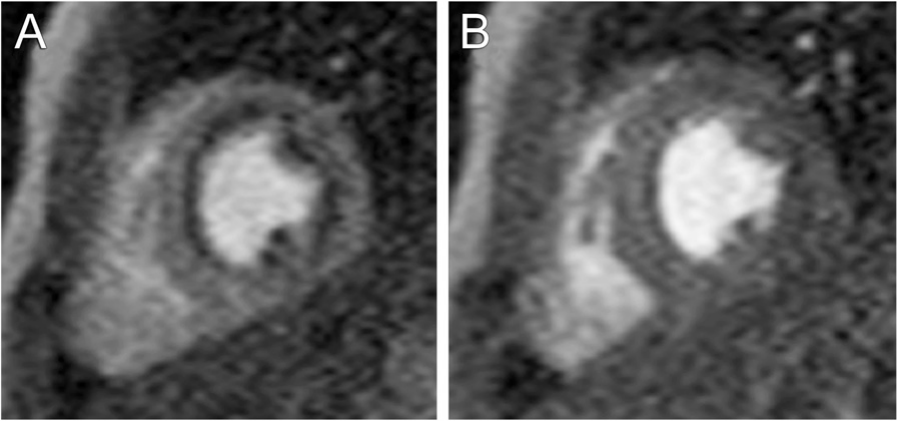
**First-pass perfusion.** Patient with anomalous origin of the left coronary artery from the pulmonary artery who underwent left coronary artery re-implantation and subsequently developed severe stenosis of the re-implanted coronary artery. First-pass perfusion images at a mid-ventricular level with adenosine stress (**A**) and at rest (**B**). Note the extensive sub-endocardial hypoperfusion of the left ventricle at stress but not at rest which indicates inducible ischemia.

Vasodilator perfusion is primarily used to assess patients for inducible myocardial ischemia (Figure 12). It relies on the principle that administration of a coronary artery vasodilator (e.g., adenosine, dipyridamole, and regadenoson) will cause a greater increase in the perfusion of myocardium supplied by normal coronary arteries than myocardium supplied by stenotic coronary arteries. Perfusion is assessed by administering an intravenous dose of a GBCA and then rapidly imaging the ventricles at multiple locations to visualize the enhancement pattern during the first transit of the contrast bolus through the myocardium. The appearance of contrast will be attenuated, both in amplitude and rate, in regions of compromised coronary blood flow. Typically, a perfusion scan is run both at rest and with vasodilator infusion in order to distinguish fixed perfusion defects (e.g., infarct) from inducible ones. As adenosine is the most commonly used vasodilator medication, the subsequent discussion will focus on this agent.

Vasodilator perfusion CMR has been shown to have similar or superior sensitivity and specificity to nuclear cardiology techniques for detecting significant coronary artery stenosis in adult patients [130-133]. Moreover, the results of perfusion CMR have been shown to have prognostic value in adults with coronary heart disease [134,135]. Reports of adenosine perfusion CMR for the assessment of coronary artery disease in children and adults with CHD have been limited to small studies [136-140]. It is increasingly being used to assess patients with chest pain, anomalous origins of the coronary arteries, and after surgeries which involve coronary artery re-implantation (e.g., the arterial switch or Ross operations). However, it is important to note that adenosine mainly induces myocardial blood flow inhomogeneities by vasodilatation and steal effects that do not necessarily represent the pathophysiology of coronary artery problems found in CHD patients. Furthermore, it is unclear whether exercise induced ischemia caused, for example, by an anomalous coronary artery coursing between the arterial roots can be detected by adenosine perfusion CMR. In some settings it may, therefore, be preferable to use other “stress” agents such as dobutamine [141] or another imaging modality in conjunction with exercise stress.

**Figure 13:**
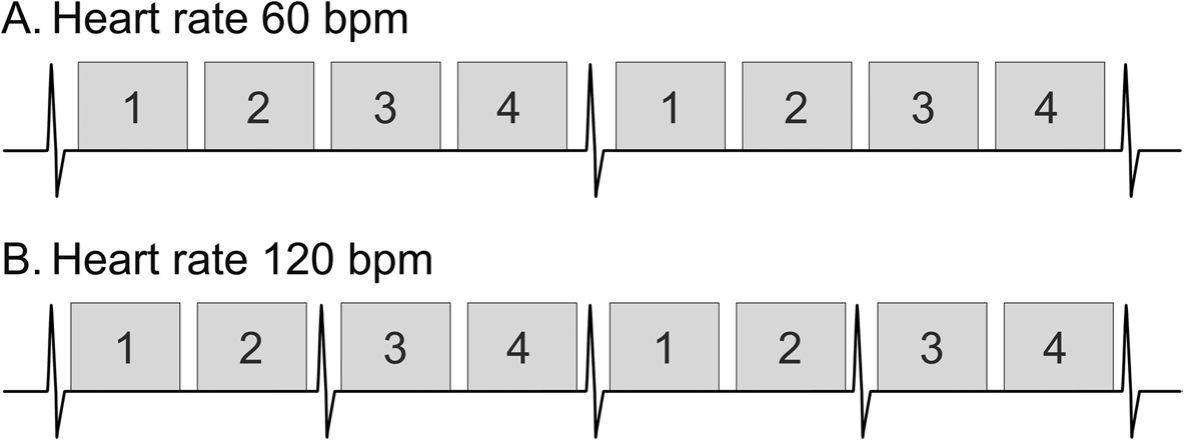
**Schematic diagram illustrating data acquisition timing in a first-pass perfusion sequence.** Each rectangle represents data acquisition to form one complete image and the numbers inside them correspond to different slice locations. At a heart rate of 60 bpm (**A**), the cardiac cycle length is long enough to allow acquisition of 4 slice locations during each beat. The slice locations are timed to different phases of the cardiac cycle, but each location is acquired repeatedly at the same phase in subsequent cycles. At a heart rate of 120 bpm (**B**), the cardiac cycle length is shorter so only two slice locations can be acquired over each beat; the other 2 locations are acquired the following beat. Note that the temporal resolution (images per unit time) is the same in **A** and **B**.

Vasodilator perfusion CMR has been performed using a variety of pulse sequences; a detailed review can be found elsewhere [142]. In brief, strong T1 contrast is provided by a preparation pulse such as inversion recovery or saturation recovery (Table 5). This is followed by fast imaging using a gradient echo, gradient echo-planar, or SSFP acquisition. Parallel imaging techniques are widely used as a means for accelerating image acquisition. These sequences create images of the heart in one heartbeat rather than acquiring data from multiple cardiac cycles to build an image as is the case for standard cine CMR. A minimum 3 short-axis slices should be prescribed to ensure coverage of all but the apical left ventricular segment. Additional short-axis or long-axis slices may also be useful. At the slower heart rates typically found in adult patients, the cardiac cycle length is long enough to allow acquisition of 3–5 slice locations during each beat (Figure 13). The slice locations are timed to different phases of the cardiac cycle but each location is acquired repeatedly at the same phase. At the higher heart rates (and shorter cardiac cycle lengths) seen in younger patients, fewer locations can be acquired in one beat. Thus, it may be advantageous to spread the imaging period for the slices over two heartbeats so that a sufficient number of locations can be acquired–each location every other beat. Spatial resolution should also be increased in smaller patients.

**Table 5:** T1-weighted saturation-recovery gradient echo for first pass myocardial perfusion

	**Infant/small child**	**Large child/adult**
**In-plane resolution (mm)**	1.5-2.0	2.0-2.5
**Slice thickness (mm)**	5-8	8-10
**Image acquisition timing**	1 R-R or 2 R-R	1 R-R

Detailed instructions on how to perform an adenosine perfusion CMR protocol have previously been published [143-145]; the basic steps follow here. Prior to undergoing adenosine perfusion CMR, the patient should be screened for contraindication to adenosine administration including second or third degree heart block, sinus node dysfunction, severe asthma or obstructive pulmonary disease, and pregnancy. Caffeine, aminophyline, and nitrate intake should be avoided on the day of the examination as these agents interfere with the action of adenosine. A specific consent procedure should be undertaken to inform the patient/guardian of potential side effects and complications such as dyspnea, flushing, headache, light-headedness, blurred vision, nausea, bronchospasm, heart block, and hypotension. It is preferable to have two intravenous lines in separate veins—one for adenosine and one for contrast agent administration—in order to avoid a bolus dose of adenosine with contrast administration. Monitoring equipment should include a continuous ECG recording and blood pressure cuff placed on the arm not being used for the adenosine infusion. Equipment and qualified personnel for resuscitation must be available.

The consensus group authors recommend performance of the vasodilator perfusion acquisition first followed by the rest acquisition with at least 15 minutes between the two so as to minimize contamination of the rest images by residual contrast given during the vasodilator infusion. An adenosine dose of 0.14 mg/kg/min is typically used though the adequacy and safety of this dose has not been thoroughly established in the pediatric age group. After the adenosine has been infusing for 3 minutes, a GBCA (0.05-0.1 mmol/kg) is rapidly injected followed by a saline flush. In adult-size patients, rates of 5 ml/s with 25 ml of flush are recommended. In children, a minimum rate of 3 ml/s through an appropriate size intravenous line and 10 ml flush is suggested. The perfusion imaging sequence is started simultaneously with the contrast agent infusion, and its scanning duration should be set to acquire approximately 60 heartbeats. In order to minimize respiratory motion artifact, breathing should be suspended as long as possible during the image acquisition. If breath-holding is not possible, the patient should be instructed to breathe shallowly. Once the imaging is completed, the adenosine infusion is discontinued. Adenosine should be terminated earlier if the patient develops persistent or symptomatic heart block, significant hypotension, or severe respiratory difficulty. An intravenous dose of aminophylline can be used to rapidly counteract the effects of adenosine. The same contrast infusion protocol and pulse sequence parameters as used in the adenosine perfusion segment should be used later for the rest perfusion imaging.

Though quantitative analysis of perfusion imaging [146] is possible using commercially available image analysis software, this process remains time-consuming to perform and is subject to technical challenges. Visual analysis by an experienced reader is usually sufficient for routine clinical practice. Interpretation is also guided by review of the cine images of ventricular function and late gadolinium enhancement (LGE) images [147]. In the absence of LGE, homogeneous enhancement in all locations at rest and with the vasodilator indicates no inducible ischemia. A region with transmural LGE and perfusion defects at rest and with the vasodilator, a so-called “fixed defect”, is also interpreted as the absence of inducible ischemia. A region that has a perfusion defect with vasodilator but is normal at rest and has no LGE is diagnostic of ischemia. Perfusion defects should be reported as either full thickness or partial thickness (sub-endocardial) with the affected region of the left ventricle described using the standard 17-segment American Heart Association model [148].

On some perfusion scans there may be a rim of reduced signal intensity in the subendocardium, which can mimic a hypoperfused area [145,149]. Compared to a true subendocardial perfusion defect, this dark rim artifact typically lasts for only a few heartbeats and then fades away. The dark rim artifact is more commonly seen with high heart rates, a more concentrated contrast bolus, and the use of a balanced SSFP rather than a gradient echo based perfusion sequences.

#### *Late gadolinium enhancement*

**Figure 14:**
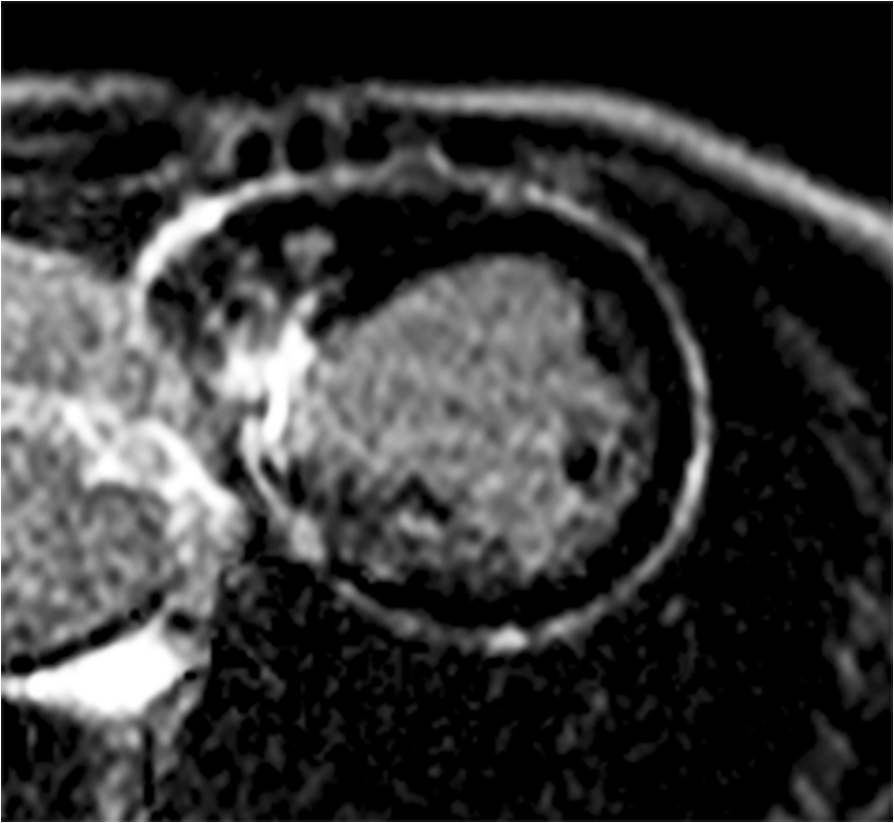
**Late gadolinium enhancement following a Fontan procedure.** Patient with tricuspid atresia and normally related great arteries who underwent a Fontan procedure. Late gadolinium enhancement image in a ventricular short-axis view showing enhancement and wall thinning of the inferior septum consistent with a chronic myocardial infarction.

**Figure 15:**
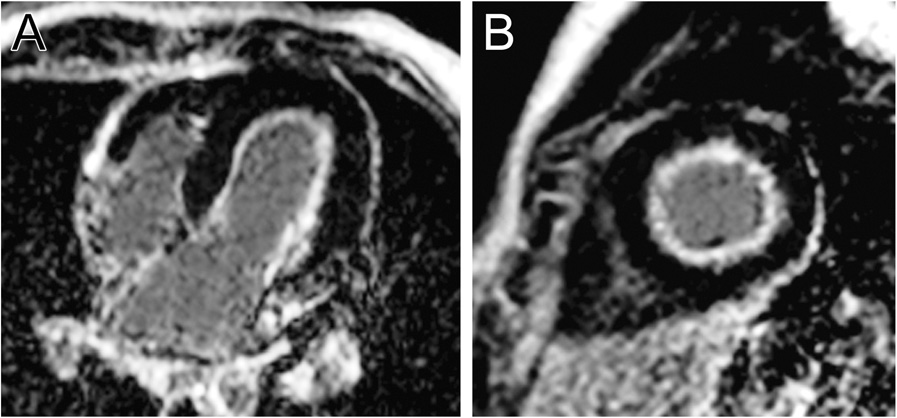
**Late gadolinium enhancement in endocardial fibroelastosis.** Patient with a history of severe congenital aortic valve stenosis who underwent balloon valvuloplasty. Late gadolinium enhancement images in 4-chamber (**A**) and short-axis views (**B**). Note the extensive subendocardial late gadolinium enhancement consistent with endocardial fibroelastosis.

LGE, also known as myocardial delayed enhancement, is a technique that detects focal regions of myocardial fibrosis and infarction. It is based on the observation that GBCA have slower washout and an increased volume of distribution in fibrotic and necrotic myocardium. Thus, such areas appear brighter than normal myocardium on LGE images. Validation studies have correlated the finding of LGE with the presence and extent of myocardial fibrosis detected by histology in animal models and in humans [150-152]. Reports in adults have demonstrated its clinical utility in acute and chronic ischemic heart disease, cardiomyopathies, myocarditis, and ventricular thrombus detection. In CHD, LGE has been described in patients with repaired tetralogy of Fallot [153-155], atrial switch operations for transposition of the great arteries [156], Fontan operations for functionally single ventricles (Figure 14) [157], congenitally corrected transposition of the great arteries [158], aortic valve stenosis [159,160], pulmonary atresia with intact ventricular septum [161], anomalous left coronary artery from the pulmonary artery after repair [162], endocardial fibroelastosis (Figure 15) [163,164], and fibrous tissue along regions of reconstruction in patients who have had surgery for CHD [165]. In the tetralogy of Fallot, atrial switch, and Fontan operation cohorts, the presence of LGE has been associated with adverse ventricular mechanics, exercise intolerance, and ventricular arrhythmias [153,155-158]. Nevertheless, the pathophysiology and prognostic impact of LGE in patients with CHD has not been fully established [166].

LGE imaging is performed 10–20 minutes following injection of 0.1-0.2 mmol/kg of a GBCA. In many imaging protocols, the contrast dose is initially used to perform a MRA. For LGE imaging, an ECG-gated 2D segmented inversion recovery gradient echo pulse sequence is typically used with data acquisition timed to coincide with the cardiac rest period to minimize motion blurring. Three-dimensional LGE sequences are available but are less well validated [167,168]. Breath-hold imaging is preferred though multiple signal average imaging, respiratory navigator gating, or single-shot imaging can be used with free-breathing. Imaging planes and slice thickness should match those used for cine imaging of the ventricles to facilitate comparison. A comprehensive examination would include LGE imaging in short-axis, LV 2, 3 and 4-chamber, and RV 3-chamber views.

In order to improve the image contrast between normal myocardium and regions of increased gadolinium concentration, an inversion pulse is incorporated into the pulse sequence. The time between the inversion pulse and image acquisition, known as the inversion time (TI), should be set to null normal myocardium. Selecting the appropriate inversion time may be facilitated by imaging iteratively with different TIs, or by the use of a TI-scout or Look-Locker sequence. Alternatively, a phase-sensitive sequence with a standardized TI based on dose, timing, and heart rate may be used [169]; such sequences provide consistent contrast between infarcted/fibrotic and normal myocardium over a wider range of TIs. Because the gadolinium concentration in normal myocardium decreases with time, the optimal TI will become longer as time elapses. Accordingly, if the time it takes to acquire all of the LGE images becomes prolonged, the TI value may need to be updated to a longer value. Initial reports suggested that the inversion time to null myocardial signal is shorter for the RV (in the usual subpulmonary position) than the LV [170,171]. However, a more recent study showed that the inversion times to null myocardium are quite similar for both ventricles, and that the apparent difference was related to insufficient spatial resolution for the thin-walled RV [172]. Thus high spatial resolution is required to properly assess for LGE on thin-walled ventricles.

**Figure 16:**
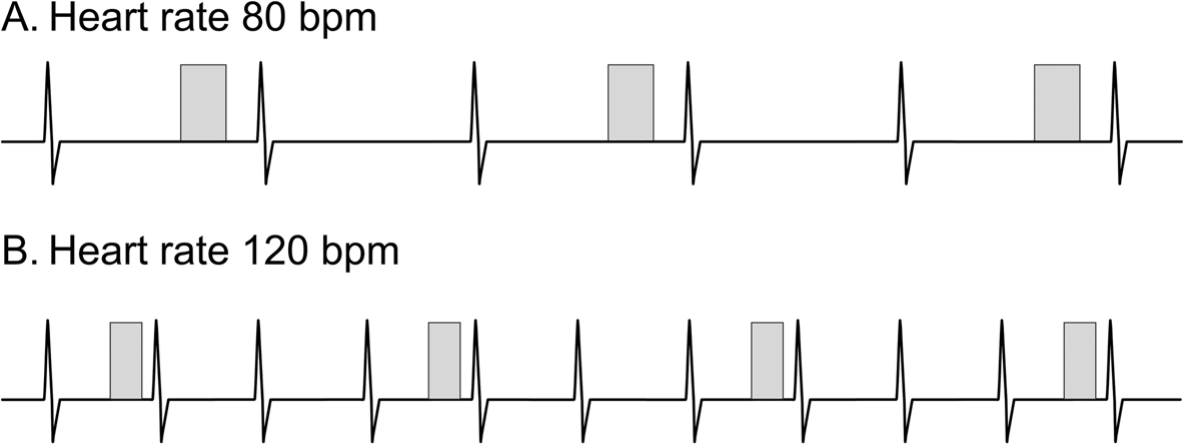
**Schematic diagram illustrating data acquisition timing in a late gadolinium enhancement sequence.** Each rectangle represents data acquisition timed to coincide with the cardiac rest period and during which a user-defined number of *k*-space lines are filled. Multiple data acquisitions and thus cardiac cycles are required to fill *k*-space and produce an image. At a heart rate of 80 bpm (**A**), data acquisition occurs every second cardiac cycle in order to allow sufficient time for recovery of longitudinal signal. At a heart rate of 120 bpm (**B**), the cardiac cycle length is shorter so the sequence is modified to acquire data every third cycle in order to maintain the same time for the recovery of longitudinal signal. In addition, the data acquisition duration is shortened to compensate for the briefer cardiac rest period associated with a faster heart rate.

Performing the LGE technique in children requires modifications to address smaller-sized ventricles and faster heart rates (Table 6). In order to ensure adequate spatial resolution, voxel size should be 1.0-1.5 mm in-plane with a thickness of 5 mm. The resulting decreased signal-to-noise ratio can be compensated for by performing two signal averages, albeit at the expense of a longer acquisition time. At higher heart rates (>100 bpm), in order to allow time for adequate longitudinal signal recovery between successive inversion pulses and avoid excessive signal loss, the interval between data acquisition should be increased from every second cardiac cycle to every third or fourth cycle (Figure 16). If the scanner software does not allow easy adjustment of the data acquisition interval, one can try manually halving the entered heart rate which may extend the scanner’s no-trigger interval and double the data acquisition interval. In addition, the data acquisition (shot) duration should be decreased by reducing the views per segment (turbo factor) to minimize blurring from faster cardiac motion at the higher heart rate.

**Table 6:** T1-weighted inversion-recovery gradient echo for late gadolinium enhancement

	**Infant/small child**	**Large child/adult**
**In-plane resolution (mm)**	1.0-1.5	1.2-2.0
**Slice thickness (mm)**	5	6-8
**Views per segment**	8-16	16-28
**Number of signal averages**	1-2	1
**Image acquisition timing**	3 R-R or 4 R-R	2 R-R
**Trigger delay**	Diastole or systole	Diastole

Although image analysis software can be used to objectively quantify the extent of enhanced myocardium [173], qualitative visual interpretation is typically performed in clinical practice. The involved cardiac segments can be reported according to the 17-segment left ventricular model [148] and a 9-segment right ventricular model [155]. In addition, it is helpful to note the extent of the wall thickness that is involved and the pattern of enhancement (e.g., subendocardial, mid-wall, subepicardial, global endocardial, septal insertion sites, and patchy). Selection of the appropriate TI is crucial for producing valid LGE images and maximizing the image intensity difference between normal and infarcted/fibrotic myocardium. If the TI is too short, the image intensity of normal myocardium will be increased and that of abnormal myocardium decreased (or even nulled) leading to gross misinterpretation. If the TI is set too long, the relative contrast between normal and abnormal myocardium will be reduced and sensitivity diminished.

Ghosting artifacts can result from regions that have a long T1 such as pericardial fluid or cerebrospinal fluid [174]. Artifact from cerebrospinal fluid can be removed by placement of a saturation band over the spinal cord. In addition, it is worthwhile to confirm the presence of enhancement by repeating LGE imaging in an orthogonal plane or by swapping the phase and frequency directions of the initial acquisition.

Finally, subendocardial, papillary muscle, and right ventricular wall enhancement may be inconspicuous when adjacent to a relatively bright blood pool. Interpreting the LGE images side-by-side with the cine images addresses this pitfall as these will provide information regarding wall thickness and papillary muscle position [174]. Moreover, performing LGE imaging later after contrast agent administration (or using a lower contrast agent dose) will lead to a lower blood pool signal and usually improved contrast against the bright enhanced regions.

### Disease-specific protocols

This section provides suggested disease-specific CMR protocols using the modules reviewed above. For conciseness, the ventriculography and LGE modules are listed once in detail at the beginning and then simply referred to in the disease-specific protocols. Similarly, the abbreviations used in the protocols are listed below for easy reference. PC CMR acquisitions are with through-plane velocity encoding unless otherwise indicated. With each condition, important data to include in reporting the examination are listed in a key reporting elements section. Both the CMR protocols and reporting elements are meant as a guide; the authors recognize that a variety of approaches may be applied to yield a comprehensive examination and report.

#### *Ventriculography module (see ventriculography section above for details)*

• Standard imaging

1. Cine CMR: left ventricular 2-chamber and 3-chamber views, right ventricular 3-chamber view, 4-chamber view (Figure 6)

2. Cine CMR: stack of contiguous slices to completely encompass both ventricles planned in a ventricular short-axis and/or axial orientation (Figure 7)

• Key reporting elements: left and right ventricular end-diastolic and end-systolic volume, stroke volume, ejection fraction, and mass; regional wall motion abnormalities

#### *LGE module (see LGE section above for details)*

• Standard imaging

1. LGE imaging in left ventricular 2-chamber and 3-chamber views, right ventricular 3-chamber view, 4-chamber view

2. LGE imaging with a stack of contiguous slices to completely encompass both ventricles planned in a ventricular short-axis or axial orientation

3. LGE imaging in an orthogonal plane to confirm LGE if present

• Key reporting elements: location, extent and thickness of LGE

#### *Coarctation of the aorta, before or after repair*

• Standard imaging

1. Cine CMR: long-axis aortic arch plane (Figure 1)

2. Ventriculography module

3. CE-MRA or 3D SSFP to image the thoracic vasculature

4. PC CMR: AAo, MPA, DAo at the level of the diaphragm

• Additional case-specific/comprehensive imaging

1. Spin echo: long-axis aortic arch plane (Figure 1)

2. Cine CMR: short-axis aortic root plane for valve morphology

3. PC CMR: quantification of collateral aortic flow by measuring proximal or just distal to the coarctation, and in the descending aorta at the level of the diaphragm

4. LGE module

• Key reporting elements: location, dimensions, and severity of aortic obstruction; arch sidedness and branching order; presence of aneurysm, dissection, or collateral vessels to the DAo; ventricular parameters including left ventricular mass; aortic valve morphology, stenosis, and regurgitation

#### *D-loop transposition of the great arteries following an arterial switch operation*

• Standard imaging

1. Ventriculography module

2. Cine CMR: long-axis of the right ventricular outflow tract plane

3. Cine CMR: oblique axial stack to image the PAs

4. CE-MRA or 3D SSFP to image the thoracic vasculature

5. PC CMR: AAo, MPA, branch PAs

• Additional case-specific/comprehensive imaging

1. 3D SSFP: coronary artery origins and proximal course

2. Vasodilator perfusion module

3. LGE module

• Key reporting elements: location and severity of AAo, MPA, and branch PA obstruction; branch PA flow distribution; proximal coronary artery patency and course; aortic root dilation; aortic and pulmonary regurgitation; ventricular parameters

#### *D-loop transposition of the great arteries following an atrial switch operation*

• Standard imaging

1. Cine CMR: axial stack from the mid-liver to the top of the aortic arch

2. Ventriculography module

3. Cine CMR: oblique planes to image the SVC and IVC pathways in long-axis

4. CE-MRA or 3D SSFP to image the thoracic vasculature, and systemic and pulmonary venous baffles (Figure 4)

5. PC CMR: AAo, MPA, branch PAs, tricuspid and mitral valves

• Additional case-specific/comprehensive imaging

1. LGE module

2. PC CMR: SVC distal to the azygous vein and IVC when systemic venous obstruction is suspected.

• Key reporting elements: location and severity of systemic and pulmonary venous pathway obstruction, atrial baffle leak, ventricular parameters, severity and mechanism of left or right ventricular outflow tract obstruction, tricuspid regurgitation, Qp/Qs, branch pulmonary artery flow distribution, SVC/IVC flow ratio as an indicator of systemic pathway obstruction

#### *Tetralogy of Fallot following complete repair*

• Standard imaging

1. Ventriculography module

2. Cine CMR: stack of images parallel to the long-axis of the right ventricular outflow tract and pulmonary valve

3. Cine CMR: oblique axial stack to image the PAs

4. CE-MRA or 3D SSFP to image the thoracic vasculature

5. PC CMR: AAo, MPA, branch PAs

• Additional case-specific/comprehensive imaging

1. LGE module

2. PC CMR: tricuspid and mitral valves

• Key reporting elements: location and severity of right ventricular outflow tract and pulmonary artery obstruction, branch pulmonary artery flow distribution, pulmonary regurgitation, atrial and ventricular septal defects, Qp/Qs, ventricular parameters including RV volumes and ejection fraction

#### *Secundum atrial septal defect*

**Figure 17:**
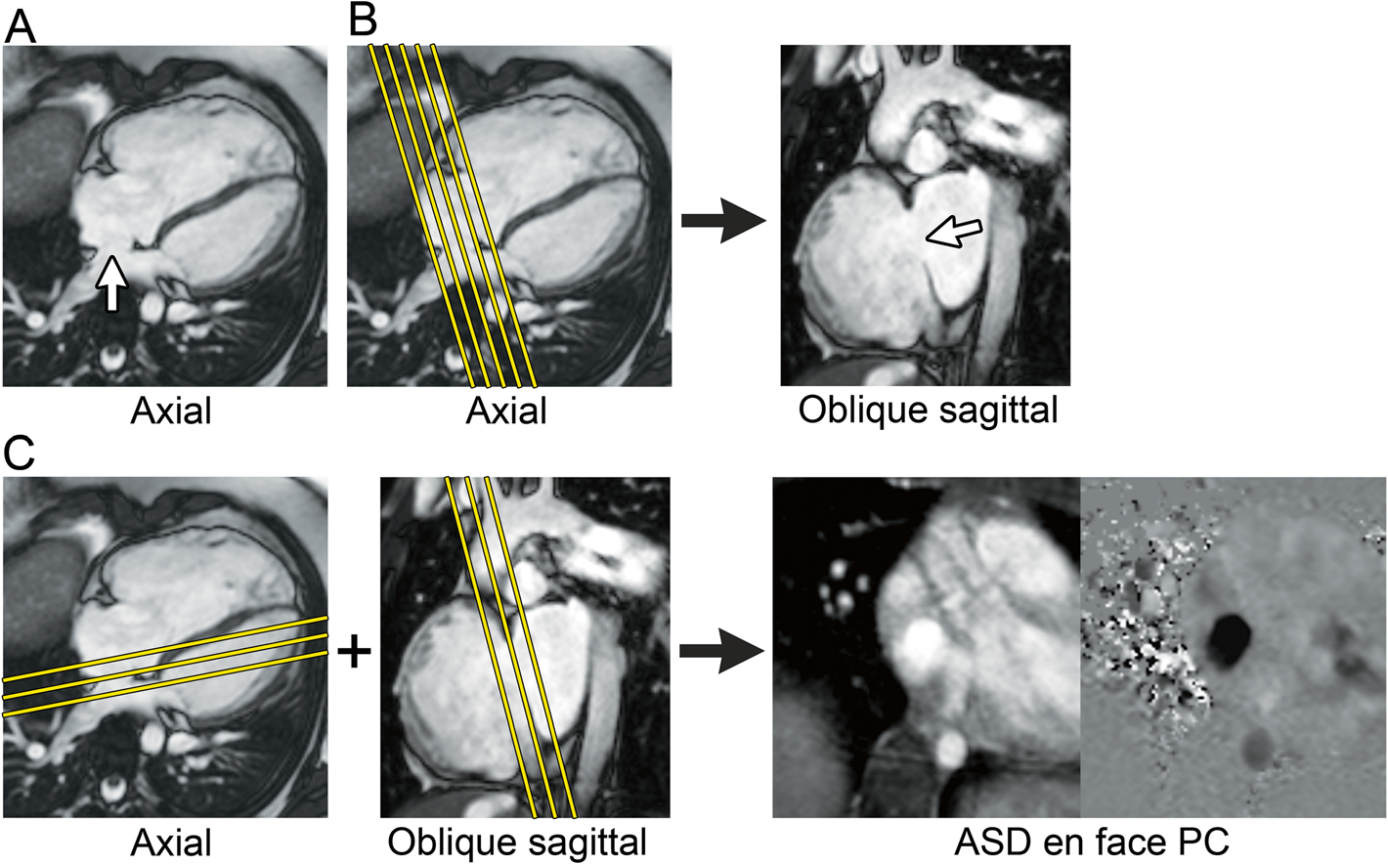
**Secundum atrial septal defect (ASD) imaging protocol. A.** Axial steady-state free precession (SSFP) image in a 10-year-old with a large secundum ASD (white arrow). **B.** The axial SSFP image is used to plan a stack of oblique sagittal SSFP images to visualize the ASD and the superior and inferior defect margins. **C.** The axial and oblique sagittal images are used together to plan a stack of phase-contrast (PC) cine images to visualize the ASD flow *en face*. This provides insight into the oval shape of the defect and may demonstrate additional ASDs.

• Standard imaging

1. Cine CMR: stack of contiguous thin slices in a 4-chamber plane to completely encompass the atrial septum

2. Cine CMR: stack of contiguous thin slices in an oblique sagittal plane perpendicular to the atrial septum to completely encompass the atrial septum (Figure 17)

3. Ventriculography module

4. PC CMR: AAo, MPA

• Additional case-specific/comprehensive imaging

1. PC CMR: 1–3 contiguous slices positioned parallel to the atrial septal plane to obtain an *en face* view of the defect (Figure 17), and with through-plane velocity encoding

2. PC CMR: stack of contiguous thin slices in a 4-chamber plane and/or in an oblique sagittal plane perpendicular to the atrial septum to completely encompass the atrial septum, and with in-plane velocity encoding in the direction of atrial septal defect flow

3. CE-MRA or 3D SSFP to image the thoracic vasculature

• Key reporting elements: number and location of the defects, defect rim measurements, ventricular parameters, Qp/Qs

#### *Partially anomalous pulmonary venous connection, before or after repair*

• Standard imaging

1. Cine CMR: axial stack from the mid-abdomen to the top of the aortic arch

2. Cine CMR: oblique plane to image the anomalous vein(s) in long-axis

3. Ventriculography module

4. CE-MRA or 3D SSFP to image the thoracic vasculature (Figure 3)

5. PC CMR: AAo, MPA, branch PAs

• Additional case-specific/comprehensive imaging

1. PC CMR: anomalous vein

• Key reporting elements: number, location, and drainage of the pulmonary veins; ventricular parameters; Qp/Qs, branch pulmonary artery flow distribution

#### *Sinus venosus septal defect*

**Figure 18:**
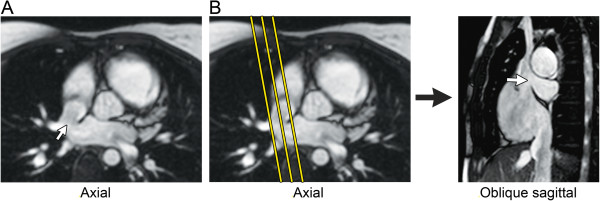
**Sinus venosus septal defect imaging protocol. A.** Axial steady-state free precession (SSFP) cine image in a 22-year-old with a large sinus venosus septal defect (white arrow). **B.** The axial SSFP image is used to plan a stack of oblique sagittal SSFP cine images to visualize the defect in an orthogonal view and assess its superoinferior dimension.

• Standard imaging

1. Cine CMR: axial stack from the mid-liver to the top of the aortic arch

2. Cine CMR: oblique sagittal plane perpendicular to the plane of the defect (Figure 18)

3. Ventriculography module

4. CE-MRA or 3D SSFP to image the thoracic vasculature

5. PC CMR: AAo, MPA

• Key reporting elements: location and size of the defect, drainage of the right pulmonary veins, ventricular parameters, Qp/Qs based on flow measurements

#### *Ebstein anomaly of the tricuspid valve*

**Figure 19:**
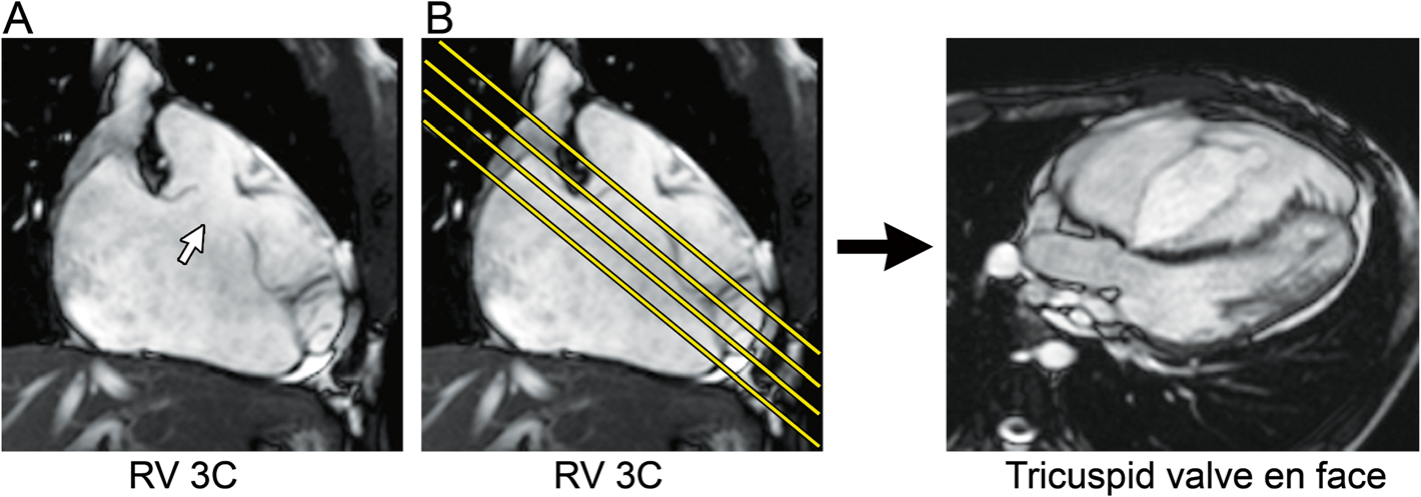
**Ebstein anomaly imaging protocol. A.** Right ventricular 3-chamber view (RV 3C) steady-state free precession cine image in a 22-year-old with Ebstein anomaly. In this example, the functional tricuspid valve plane is displaced and inflow is directed into the right ventricular outflow tract (white arrow). **B.** The RV 3C image is used to plan a stack of cine images to visualize the displaced tricuspid valve orifice *en face* for anatomic assessment or flow quantification.

• Standard imaging

1. Cine CMR: axial stack from the diaphragm to the top of the aortic arch

2. Ventriculography module with multiple contiguous slices in the right ventricular 3-chamber view and in the 4-chamber view

3. PC CMR: AAo, MPA, tricuspid valve, mitral valve

• Additional case-specific/comprehensive imaging

1. Cine CMR: contiguous stack oriented parallel to the functional tricuspid valve plane to visualize the valve en face (Figure 19)

2. CE-MRA or 3D SSFP to image the thoracic vasculature

• Key reporting elements: description of tricuspid valve morphology, tricuspid regurgitation and stenosis, pulmonary stenosis, ventricular parameters, presence of an atrial septal defect, Qp/Qs based on flow measurements

#### *Functional single ventricle following a stage 1 or 2 palliation*

• Standard imaging

1. Cine CMR: axial stack from the mid-liver to the top of the aortic arch

2. Ventriculography module

3. CE-MRA or 3D SSFP to image the thoracic vasculature and surgical shunts

4. PC CMR: AAo, native MPA, branch PAs

• Additional case-specific/comprehensive imaging

1. LGE module

2. Cine CMR: long-axis aortic arch plane

3. Spin echo: axial plane to image the branch PAs and aortopulmonary shunt

4. PC CMR: SVC, IVC, tricuspid and mitral valves, DAo at the level of the diaphragm, pulmonary veins

• Key reporting elements: shunt, branch PA, pulmonary vein, and aortic arch obstruction; ventricular parameters; valve regurgitation; aortopulmonary collaterals; venous collaterals

#### *Functional single ventricle following a Fontan operation*

• Standard imaging

1. Cine CMR: axial stack from the mid-liver to the top of the aortic arch

2. Cine CMR: coronal or oblique stack to image the Fontan baffle in long-axis

3. Ventriculography module

4. CE-MRA or 3D SSFP to image the thoracic vasculature

5. PC CMR: AAo, native MPA, branch PAs, SVC, IVC

• Additional case-specific/comprehensive imaging

1. Cine CMR: long-axis aortic arch plane

2. LGE module

3. PC CMR: tricuspid and mitral valves, pulmonary veins

•Key reporting elements: Fontan pathway, SVC, IVC, branch PA, pulmonary vein, and aortic arch obstruction; Fontan baffle defects; ventricular parameters; valve regurgitation; aortopulmonary collaterals

## Abbreviations

2C: 2-chamber; 2D: 2-dimensional; 3C: 3-chamber; 4C: 4-chamber; 3D: 3-dimensional; AAo: Ascending aorta; ASD: Atrial septal defect; CE-MRA: Contrast-enhanced magnetic resonance angiography; CHD: Congenital heart disease; CMR: Cardiovascular magnetic resonance; DAo: Descending aorta; ECG: Electrocardiogram; GBCA: Gadolinium-based contrast agents; LGE: Late gadolinium enhancement; LV: Left ventricle; IVC: Inferior vena cava; MPA: Main pulmonary artery; MR: Magnetic resonance; MRA: Magnetic resonance angiography; NSF: Nephrogenic systemic fibrosis; PAs: Pulmonary arteries; PC CMR: Velocity-encoded phase-contrast cine CMR; Qp/Qs: Pulmonary-to-systemic flow ratio; RV: Right ventricle; RVOT: Right ventricular outflow tract; SA: Short-axis; SSFP: Steady-state free precession; SVC: Superior vena cava; TE: Echo time; TFE: Turbo field echo; TI: Inversion time; TR: Repetition time; venc: Velocity encoding range.

## Competing interests

The authors declare that they have no competing interests.

## Authors’ contributions

SF drafted and conceived of the review, designed the initial structure of the manuscript, coordinated author communication, and revised the authors’ contributions to produce the draft version of the manuscript; TC drafted the ventriculography section; GFG drafted the contrast-enhanced MRA and 3D SSFP sections; MMS provided extensive editorial assistance; AMT drafted of the vasodilator perfusion section; ERVB drafted the sedation section; SJY drafted the blood velocity and flow measurement section; AJP drafted the LGE and patient preparation sections, and revised and synthesized all of the authors’ contributions to produce the final version of the manuscript. All authors contributed substantially to the design of the review, critically revised the manuscript for important intellectual content, and read and approved the final manuscript.
